# Cystic fibrosis pathogens persist in the upper respiratory tract following initiation of elexacaftor/tezacaftor/ivacaftor therapy

**DOI:** 10.1128/spectrum.00787-24

**Published:** 2024-06-25

**Authors:** Yasmin Hilliam, Catherine R. Armbruster, Glenn J. Rapsinski, Christopher W. Marshall, John Moore, Junu Koirala, Leah Krainz, Jordan R. Gaston, Vaughn S. Cooper, Stella E. Lee, Jennifer M. Bomberger

**Affiliations:** 1 Department of Microbiology and Molecular Genetics, University of Pittsburgh, Pittsburgh, Pennsylvania, USA; 2 Department of Biological Sciences, Marquette University, Milwaukee, Wisconsin, USA; 3 University of Pittsburgh Medical Center, Pittsburgh, Pennsylvania, USA; University of Arkansas for Medical Sciences, Little Rock, Arkansas, USA

**Keywords:** cystic fibrosis, CFTR, modulator therapy, microbiome, sinus, chronic rhinosinusitis

## Abstract

Elexacaftor/tezacaftor/ivacaftor (ETI) therapy has revolutionized the treatment of cystic fibrosis (CF) for most affected individuals but the effects of treatment on sinus microbiota are still unknown. Changes to the airway microbiota in CF are associated with disease state and alterations to the bacterial community after ETI initiation may require changes to clinical management regimens. We collected sinus swab samples from the middle meatus in an observational study of 38 adults with CF and chronic rhinosinusitis (CRS) from 2017 to 2021 and captured the initiation of ETI therapy. We performed 16S and custom amplicon sequencing to characterize the sinus microbiota pre- and post-ETI. Real-time quantitative PCR (RT-qPCR) was performed to estimate total bacterial abundance. Sinus samples from people with CF (pwCF) clustered into three community types, dependent on the dominant bacterial organism: a *Pseudomonas*-dominant, *Staphylococcus*-dominant, and mixed dominance cluster. Shannon’s diversity index was low and not significantly altered post-ETI. Total bacterial load was not significantly lowered post-ETI. *Pseudomonas* spp. abundance was significantly reduced post-ETI, but eradication was not observed. *Staphylococcus* spp. became the dominant organism in most individuals post-ETI and we showed the presence of methicillin-resistant *Staphylococcus aureus* (MRSA) in the sinus both pre- and post-ETI. We also demonstrated that the sinus microbiome is predictive of the presence of *Pseudomonas* spp., *Staphylococcus* spp., and *Serratia* spp. in the sputum. *Pseudomonas* spp. and *Staphylococcus* spp., including MRSA, persist in the sinuses of pwCF after ETI therapy, indicating that these pathogens will continue to be important in CF airway disease management in the era of highly effective modulator therapies (HEMT).

**IMPORTANCE:**

Highly effective modulator therapies (HEMT), such as elexacaftor/tezacaftor/ivacaftor (ETI), for cystic fibrosis (CF) have revolutionized patient care and quality of life for most affected individuals. The effects of these therapies on the microbiota of the airways are still unclear, though work has already been published on changes to microbiota in the sputum. Our study presents evidence for reduced relative abundance of *Pseudomonas* spp. in the sinuses following ETI therapy. We also show that *Staphylococcus* spp. becomes the dominant organism in the sinus communities of most individuals in this cohort after ETI therapy. We identified methicillin-resistant *Staphylococcus aureus* (MRSA) in the sinus microbiota both pre- and post-therapy. These findings demonstrate that pathogen monitoring and treatment will remain a vital part of airway disease management for people with cystic fibrosis (pwCF) in the era of HEMT.

## INTRODUCTION

The life expectancy of people with cystic fibrosis (pwCF) has steadily increased over several decades through groundbreaking research and improvements in medical treatment, however, chronic bacterial infections in the airways, which can lead to lung failure, remain the leading cause of mortality (1). *Pseudomonas aeruginosa* and *Staphylococcus aureus* are known to be important cystic fibrosis (CF) pathogens that lead to reduced lung function and increased pulmonary exacerbations (2–10) but with improvements in culture-free detection it has become clear that there are complex communities of microorganisms, including viruses and fungi, occupying the airways of pwCF throughout their lives. Microbiome profiling has uncovered important links between community members, structure, and dysbiosis in several disease states in the airway including asthma, chronic obstructive pulmonary disease, and non-CF bronchiectasis (11–14). In CF decreases in bacterial diversity in the lung have been shown to correlate with reduced lung function and disease progression (15).

The introduction of highly effective modulator therapies (HEMT) has revolutionized CF care for a majority of people. Elexacaftor/tezacaftor/ivacaftor (ETI) triple therapy is approved by the US Food and Drug Administration for use in pwCF over 2 years of age with at least one copy of F508del, accounting for approximately 90% of pwCF (16). Studies have shown marked improvements in clinical outcomes and quality of life measurements for pwCF taking ETI (17–19). The airway milieu during ETI treatment is significantly altered (20) and there have been several studies showing reductions in bacterial load and pathogen abundance, along with increases in commensal flora in the CF lung following modulator initiation (21, 22). Research into the long-term effects of ETI on both the host and microbiota is ongoing; yet the large-scale, multi-center studies investigating alterations to the airway microbiology are focused on the lower airway, with little work being carried out on upper airway samples.

Attention to sinus disease has been limited in CF, despite the prevalence of chronic rhinosinusitis (CRS) as a comorbidity because lung disease is the leading cause of morbidity and mortality in pwCF (1). CF-CRS has serious implications for health and quality of life. It is characterized by inflammation of the sinonasal epithelium and, in pwCF, is commonly accompanied by the formation of nasal polyps (23). CF-CRS manifests clinically as pressure in the sinuses, nasal congestion, and decreased sense of smell. Viscous mucus in the sinus contributes to decreased mucociliary clearance and provides an ideal environment for the establishment of chronic bacterial infection in the sinus. Several studies have provided evidence that bacteria are aspirated from the sinus into the lung (24–26) and there is evidence that strains colonizing the sinuses may become adapted to the host environment in the sinuses prior to colonizing the lung (27). Many pwCF undergo functional endoscopic sinus surgery (FESS) during which part of the sinus structures are removed under direct endoscopic visualization to improve airflow and mucus clearance from the sinus cavities. FESS is a minimally invasive procedure but can lead to alterations in the sinus environment that alter the microbiota of the sinus in pwCF (28, 29). The unified airway theory posits that the upper and lower airway form a single organ with shared physiological and immunologic traits (30, 31). There are undoubtedly similarities and shared traits between the microbiota of the sinus and lungs, particularly given the bidirectional movement through the airway as a result of inhalation and mucociliary clearance. However, there are observed differences in these niches as well in both host environments (32) and microbial community profiles (33, 34).

Key differences between the sinus and lung physiology and immune milieu may contribute to observed differences between the microbiota at each site (32). However, in the era of HEMT, many pwCF are no longer able to spontaneously expectorate sputum for sampling at clinic visits. This translates into a loss of important data for clinicians in determining the best course of treatment for ongoing airway infections. Early studies have indicated that bacterial load and pathogen abundance are reduced post-ETI but remain elevated when compared to healthy controls (21, 22, 28). The gold standard for microbial sampling of the lungs is through bronchoalveolar lavage but this is not routinely performed during CF clinic visits. Identifying minimally invasive alternative sampling techniques for pathogen monitoring in pwCF is imperative and it is useful to determine if endoscopically guided sinus sampling may be diagnostic of pathogens persisting in the lungs.

In this study, we aimed to capture changes in the bacterial community of the sinuses in pwCF after the initiation of ETI therapy. Through 16S rRNA amplicon sequencing and RT-qPCR, we sought to identify differences in overall bacterial load pre- and post-ETI and any changes in microbial diversity. We further aimed to identify taxa that were differentially abundant in the sinuses post-ETI and determine if the bacterial community dynamics were altered to more closely resemble healthy sinus microbiota, such as increased alpha diversity and reduced pathogen burden (35, 36). Through comparison of sinus and sputum samples from our cohort, we aimed to quantify the utility of sinus samples in predicting sputum microbiota composition. Here, we show that the community structure of the CF sinus microbiota changes post-ETI, driven mostly by reductions in *Pseudomonas* spp. abundance and increases in *Staphylococcus* spp. abundance. We demonstrate the presence and persistence of methicillin-resistant *S. aureus* (MRSA) in the sinuses after ETI initiation. Finally, we show that sinus sampling can predict the presence of important CF pathogens in the sputum.

## RESULTS

### Study cohort

Subjects were enrolled in an observational study at the University of Pittsburgh Medical Center Adult CF Sinus Clinic following an IRB-approved protocol (STUDY19100149). We collected upper (sinus swab) and lower (expectorated sputum) respiratory tract samples from subjects attending the adult CF treatment center whilst receiving treatment for CRS. Sinus samples were collected by endoscopically guided, sheathed swabs to prevent contact with and contamination from the nares. Subjects enrolled in the study had previously received FESS to relieve CRS symptoms. Over 42 months we collected longitudinal samples from 38 subjects for microbiota analysis. To examine the relationship between ETI treatment and bacterial community composition in the CF sinus we identified 14 subjects who provided at least one sinus sample prior to initiating ETI treatment and at least one sinus sample after ETI initiation. Five subjects were not prescribed ETI during this study and 19 subjects who were prescribed ETI during the study but did not provide any further sinus samples post-ETI initiation. In total, we analyzed 65 sinus samples (pre-ETI *n* = 44, post-ETI *n* = 21) to compare outcomes pre- and post-ETI initiation (Table 1; Fig. S1).

**TABLE 1:** Cohort table for this study

Total subjects enrolled = 38		
Median age at enrollment (years; range)		29.95 (21.21–49.72)
Male (%)		17 (45%)
Race (%)		
White		37 (97%)
Other		1 (3%)
Cystic fibrosis transmembrane conductance regulator (CFTR) genotype (%)		
ΔF508 homozygous		21 (55%)
ΔF508/other		15 (40%)
Unknown		2 (5%)
Cystic fibrosis related diabetes (CFRD) (%)		22 (58%)
Median follow-up period (weeks; range)		84 (0–179)
ETI prescription status (%)		
Not prescribed		5 (13%)
Prescribed, no sinus follow-up		19 (50%)
Prescribed, ≥1 sinus follow-up		14 (37%)
Sinus samples		Total = 65
Pre-ETI		44
Post-ETI		21
Paired sinus and sputum samples		Total = 108

### Sinus microbiota are individualized and are not stable over time

**Fig 1:**
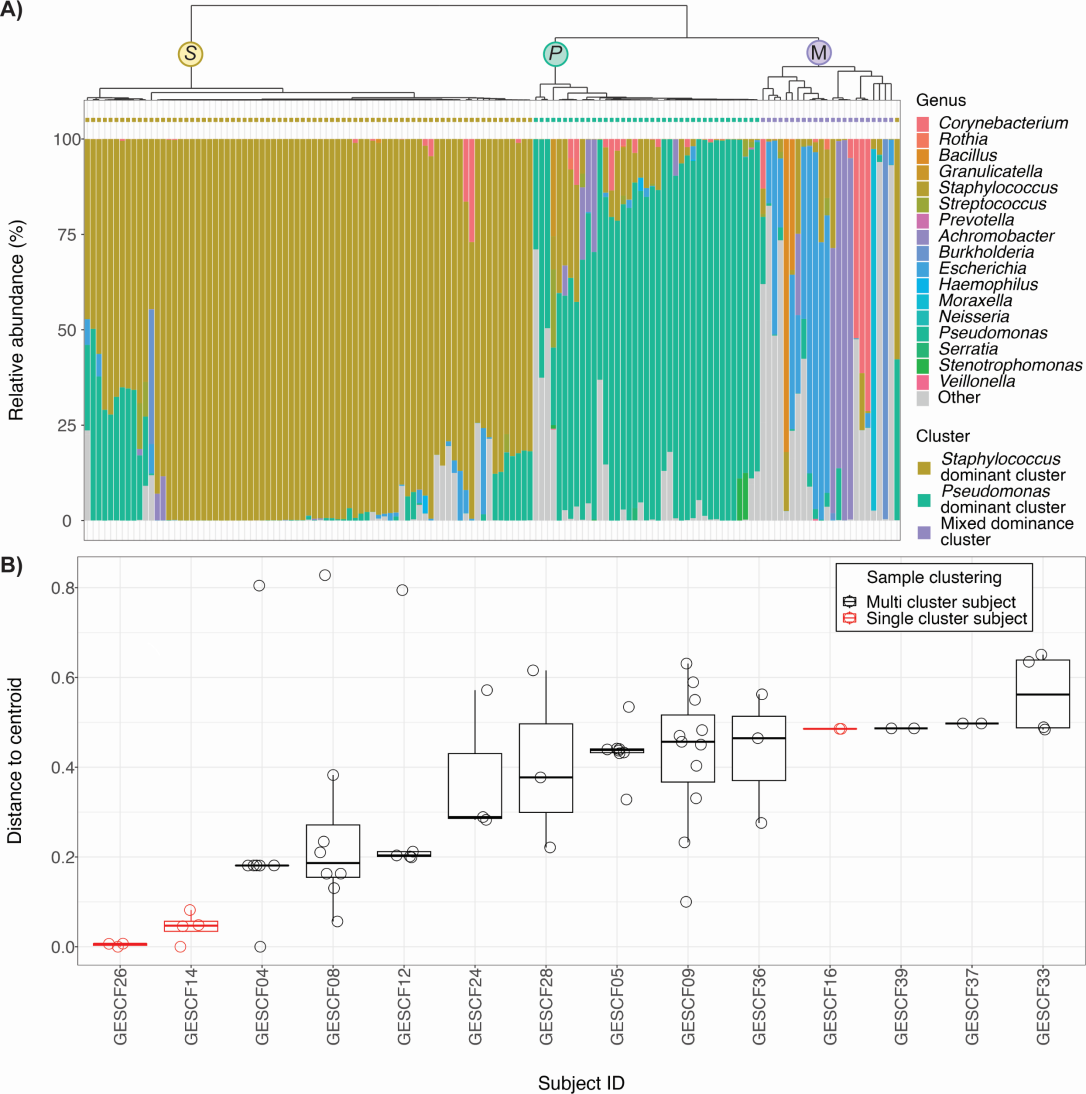
(A) Sinus samples from pwCF who provided ≥1 pre- and post-ETI samples form three clusters based on community composition and are driven by the dominant genera observed in each sample. Each stacked bar represents the relative abundance of taxa in a single sample and the colored bar at the top of the Y-axis represents the cluster of samples beneath it. (B) Centroid distances derived from the Morisita-Horn distance matrix vary within and between subjects. Subjects with samples that fall within different clusters tend to have higher centroid distances between sinus samples. Each point represents the calculated distance from the per-subject centroid per sample. Boxplots and points in red indicate subjects whose samples all fell within one cluster, those in black represent subjects whose samples fell in multiple clusters.

We first examined the microbial community composition of each sample and compared it between subjects to determine if there is a shared community structure common to the sinuses of pwCF. Through hierarchical clustering we identified three clusters of samples determined by their observed dominant genera, giving rise to a *Staphylococcus*-dominant cluster, a *Pseudomonas*-dominant cluster, and a mixed dominance cluster (Fig. 1A) (37, 38). To assess the variability of microbiota within subjects and how that variability compares between subjects, we measured per sample variation from the subject average by calculation of centroid distances (37) (Fig. 1B). We observed variations in community composition of the sinus microbiota both within and between subjects over time. Increased centroid distance was associated with subjects with samples from multiple clusters over time, as determined by the linear mixed-effect model (39, 40) (*P* = 0.0146), suggesting that the instability of CF airway microbiota over time is associated with changes in the dominant organism at the time of sampling.

### Most patients experience a change in microbiota composition post-ETI

**Fig 2:**
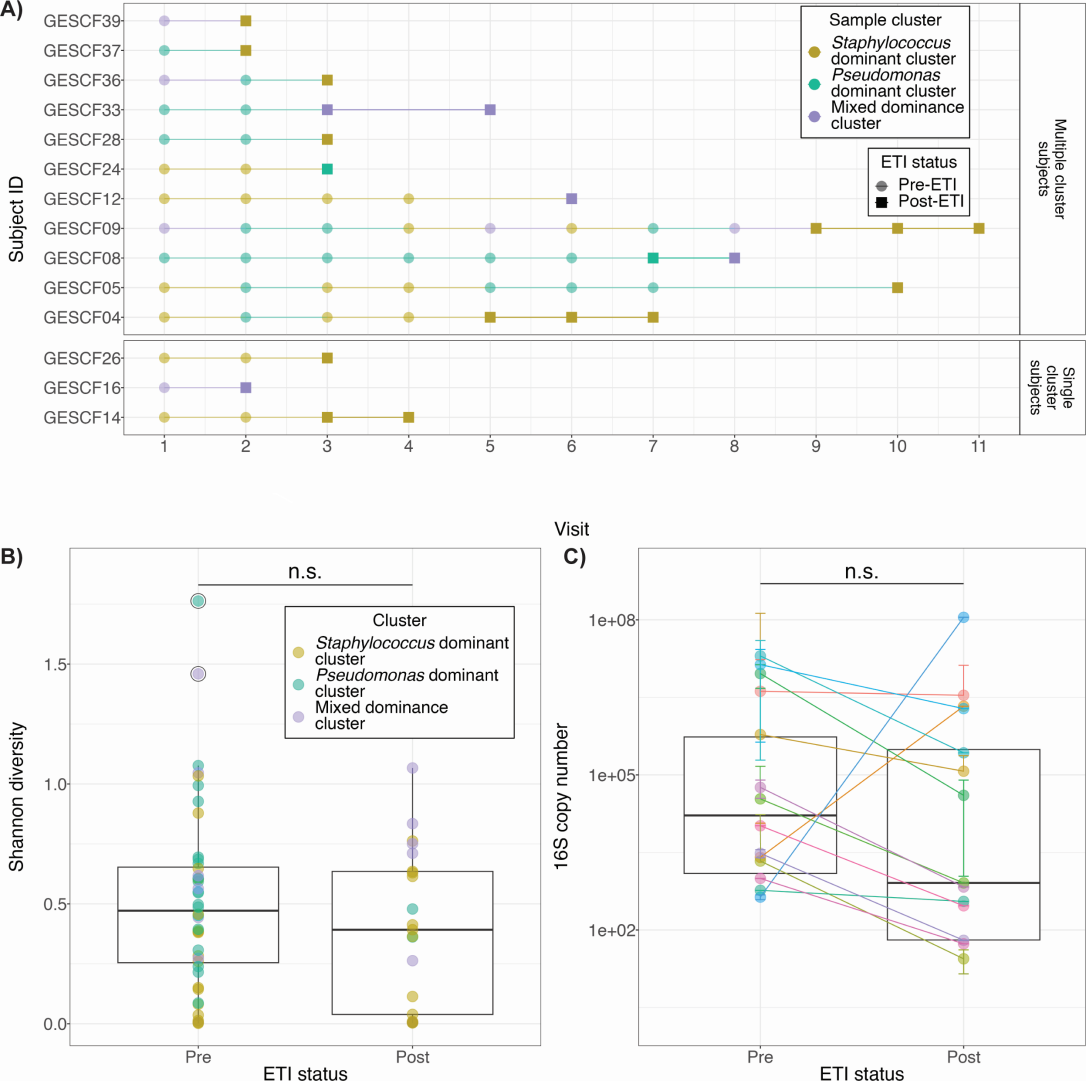
(A) Most subjects experience a change in sinus microbiota post-ETI, with a greater proportion of samples falling in the *Staphylococcus*-dominant cluster post-ETI. Shaded circles represent pre-ETI samples and solid squares represent post-ETI samples, with colors representing the three identified clusters. (B) There is no observed change in overall Shannon diversity post-ETI as determined by the use of a linear mixed-effect model accounting for multiple measures per patient. Colored circles represent individual samples and are colored by the cluster to which each sample was assigned. Black circles around a colored point indicate outliers. Boxplots represent median and interquartile ranges for all samples pre- and post-ETI. (C) There is a trend toward decreased total bacterial abundance as measured by 16S rRNA copy number post-ETI but there is no significant difference between bacterial abundance pre- and post-ETI across all subjects as determined by a linear mixed-effect model accounting for multiple measures per subject. Colored circles and lines represent the mean estimated total bacterial abundance for individual subjects pre- and post-ETI, with error bars to indicate standard deviation from the mean. Boxplots represent median and interquartile ranges for all samples pre- and post-ETI.

We next examined the relationship between sample clustering over time and ETI status to identify overall changes in sinus microbiota after ETI initiation. Our cohort included 14 subjects who had paired pre- and post-ETI sinus samples. Seventy-nine percent of subjects experienced a change in sample cluster post-ETI versus pre-ETI (Fig. 2A). Interestingly 64% of subjects experiencing a change in cluster underwent a shift in microbiota that moved their samples to the *Staphylococcus*-dominant cluster. The overall effect observed was an increase in the proportion of samples in the *Staphylococcus*-dominant and mixed dominance clusters, and a decrease in the proportion of samples falling in the *Pseudomonas*-dominant cluster. Despite an apparent shift in microbiota in a majority of subjects post-ETI, there was no significant change in Shannon diversity (39, 40) in the sinuses (Fig. 2B), which remains low (median pre-ETI = 0.451; median post-ETI = 0.392). Most post-ETI samples had reduced total bacterial abundance (Fig. 2C), but the difference is not statistically significant, as determined by the linear mixed-effect model (39, 40). These results suggest that despite a reduction in bacterial load post-ETI, pwCF are still experiencing single taxa dominance and low microbial diversity post-ETI.

We also sequenced the internal transcribed spacer (ITS) region to identify fungal species in sinus samples pre- and post-ETI. Only eight of 14 patients had both pre- and post-ETI samples in which fungal reads were detectable and the number of taxa identified per sample ranged from 1 to 5, leaving us underpowered to conclude changes to the fungal community in the sinuses of pwCF post-ETI. The most abundant and prevalent fungal genus identified was *Malassezia* spp. and we did not detect any *Aspergillus* spp. among these subjects (Fig. S3).

### *Pseudomonas* spp. reduced but not eradicated in the sinuses post-ETI

**Fig 3:**
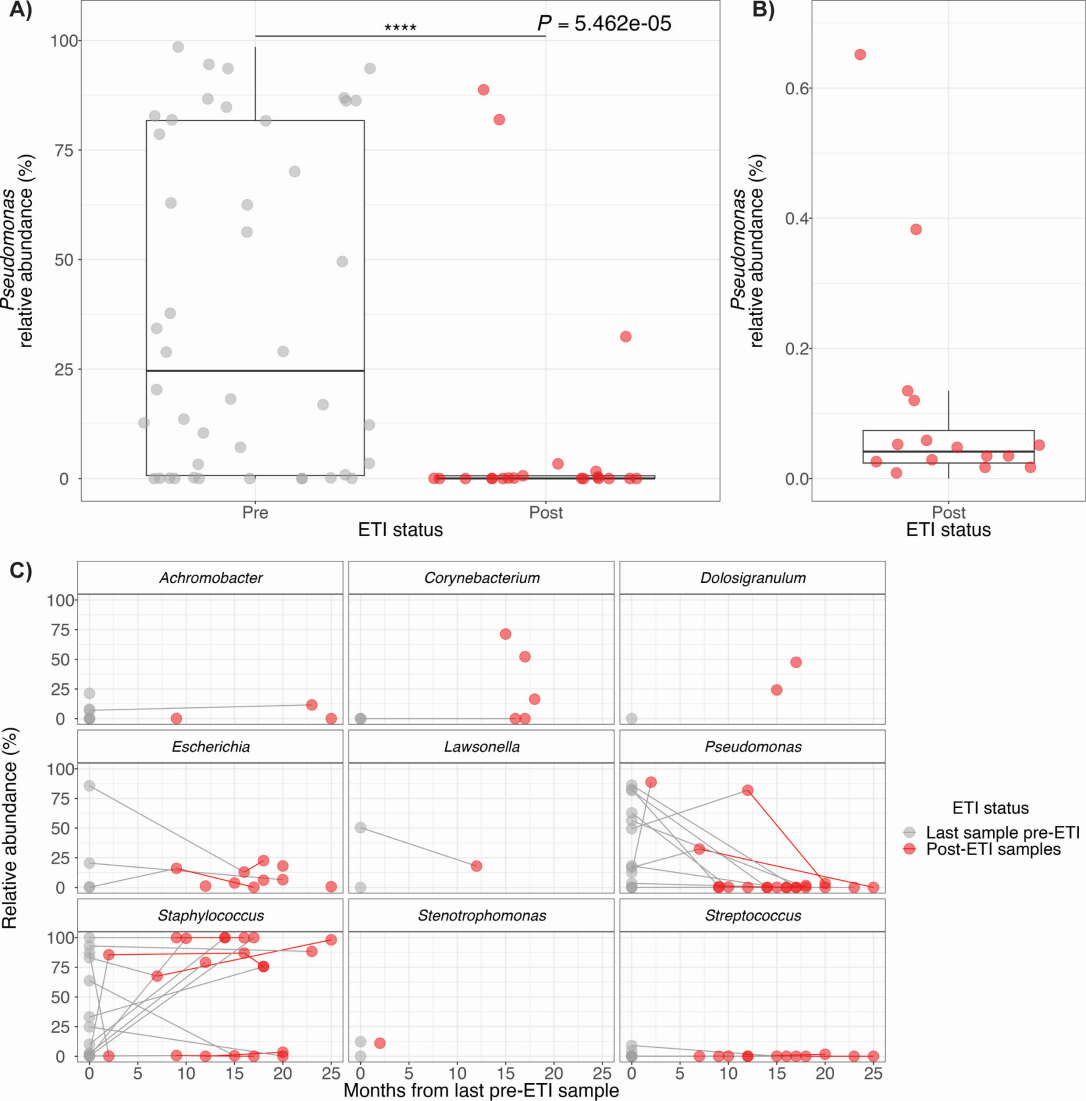
(A) *Pseudomonas* relative abundance is significantly reduced (*P* = 5.462 × 10^−5^) in the sinus following initiation of ETI therapy. Multivariable linear modeling using MaAsLin2 identified *Pseudomonas* spp. as the only differentially abundant taxa across patients pre- and post-ETI. Grey points represent all pre-ETI samples from all subjects and red points indicate all post-ETI samples from all subjects. Boxplots represent median and interquartile ranges for all samples pre- and post-ETI. (B) *Pseudomonas* spp. relative abundance does not drop to 0% in any but one sample post-ETI indicating that there is persistence in the sinuses. Red points indicate all post-ETI samples from all patients. Boxplot represents median and interquartile ranges for all samples post-ETI. (C) *Staphylococcus* spp. is the only genus where a trend toward increased relative abundance in the months following ETI initiation is observed. Gray points represent the last available pre-ETI sample per subject and red points indicate all subsequent post-ETI samples per subject. Points joined by a line were obtained from the same subject.

We observed changes in how subject samples clustered pre- and post-ETI, indicating that there were major shifts in community composition following ETI initiation. We next sought to identify differentially abundant taxa responsible for changes to the sinus microbiota post-ETI. Data were analyzed using MaAsLin 2 (41) to perform multivariable linear modeling, accounting for repeated measures, to identify differentially abundant and prevalent taxa pre- and post-ETI (Table S1). *Pseudomonas* spp. was identified as the only significantly differentially abundant genus across subjects following initiation of ETI therapy (Fig. 3A). In samples where *Pseudomonas* spp. were detected, pre-ETI median relative abundance was 28.9% (mean = 40.2%, range = 0.009%–98.5%); post-ETI median relative abundance was 0.05% (mean = 10.5%, range = 0.008%–88.8%). Despite the reduction of *Pseudomonas* spp. relative abundance post-ETI, it remained detectable by 16S rRNA sequencing at low levels in all 14 subjects (Fig. 3B). *Pseudomonas* spp. was undetectable in only one post-ETI sample from subject GESCF09, but was subsequently detected at low abundance (<2%) in later samples provided by this individual. These data provide evidence for a significant reduction, but not eradication of *Pseudomonas* spp. in the sinuses of pwCF in the months following initiation of ETI therapy.

### *Staphylococcus* spp. relative abundance increases to dominance in many patients post-ETI

To investigate changes in dominant organisms in patients following initiation of ETI, we plotted the relative abundance of sinus taxa over time. We used a subset of data to include the last available pre-ETI sample from each subject and subsequent post-ETI samples. Interrogation of the last available pre-ETI sinus sample and all available post-ETI samples per subject for each genus revealed variable dynamics of relative abundance shifts per genera in the months following initiation of ETI (Fig. 3C; Fig. S4). Post-ETI we observed increased or sustained high relative abundance of *Staphylococcus* spp. in the sinus, a pattern not observed for any other genera (Fig. S4). When increased *Staphylococcus* spp. relative abundance was observed post-ETI the mean increase was 59.3%; across all subjects (including those with decreased *Staphylococcus* relative abundance post-ETI) the average change in abundance was +18.2%. In the months after the initiation of ETI therapy, many subjects experienced a shift in microbiota that resulted in a *Staphylococcus*-dominated community.

### Methicillin-resistant *S. aureus* is present in the sinuses pre-ETI and persists in high abundance post-ETI

Increased relative abundance of *Staphylococcus* spp. in the sinuses post-ETI could be indicative of external influences (i.e., inhalation and repopulation of commensal Staphylococci from the nares) or could be a result of establishment or expansion of pathogenic strains already extant in the sinus community. Understanding the species-level composition of the sinus microbiota is important in determining future paths for research or treatment. Amplicon sequencing using custom oligos allowed for the identification of samples containing *S. aureus*, *Staphylococcus epidermidis*, and MRSA.

**Fig 4:**
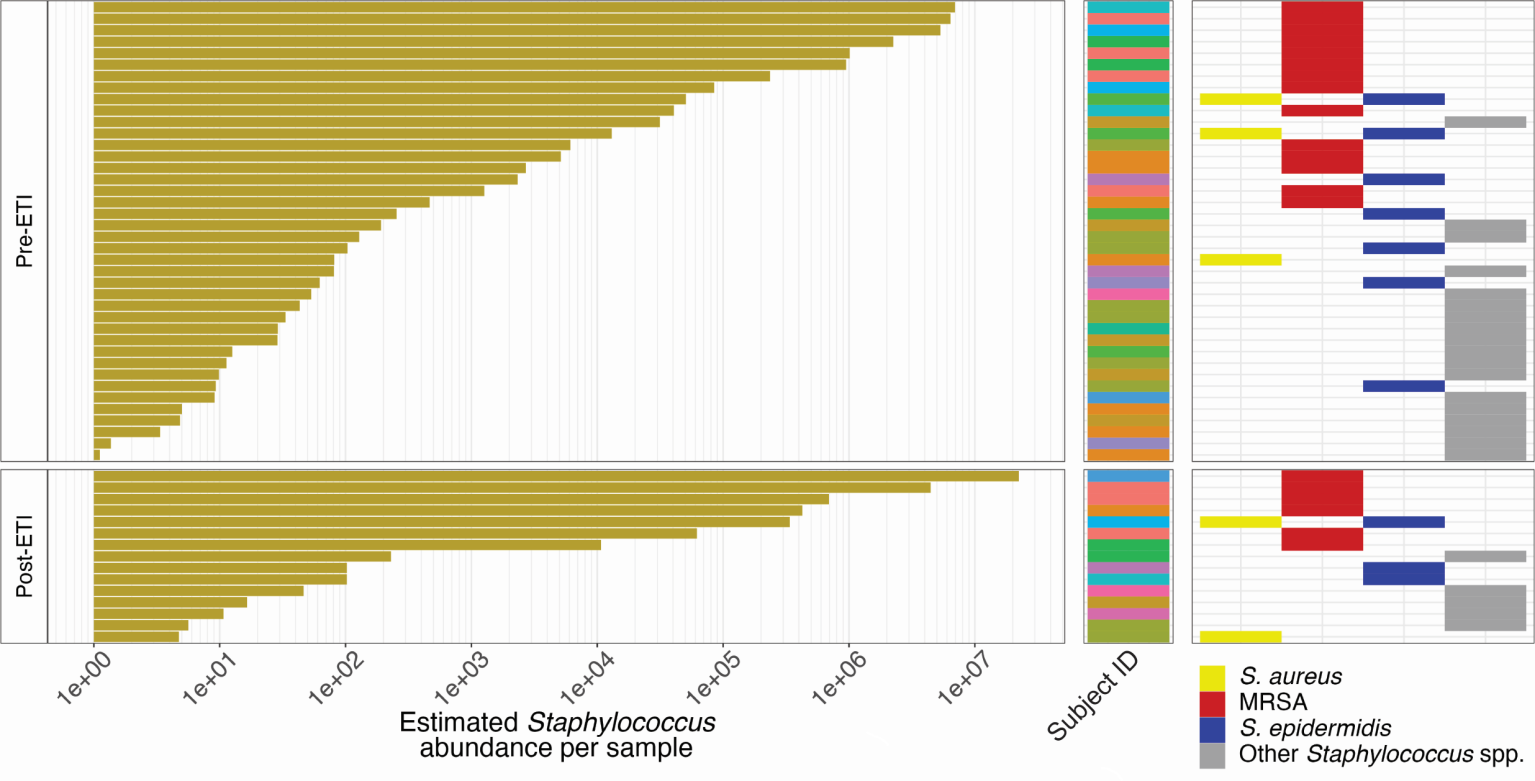
MRSA is present in the sinuses in high abundance pre-ETI and persists at high abundance post-ETI. Yellow bars indicate the estimated total *Staphylococcus* spp. abundance per sample. Colored stripes indicate which subject samples were obtained from. Colored tiles indicate positivity as determined by species/strain-specific amplicon read counts.

We plotted estimated *Staphylococcus* abundance per sample alongside a plot indicating the presence of strain/species-specific amplicons in each sample. MRSA (red) is present in almost all sinus samples with high *Staphylococcus* spp. abundance, both pre- and post-ETI. The middle column of the plot indicates which subject each sample was collected from and demonstrates that the high abundance of *Staphylococcus* and the presence of MRSA in these samples is not driven by samples from a single subject (Fig. 4). The presence of MRSA both pre- and post-ETI suggests that alterations to the host environment by ETI treatment are not detrimental to pathogenic Staphylococci and suggest that, in the absence of high numbers of *Pseudomonas* spp.*,* they may be able to expand to become the dominant organism in the sinuses.

### Sinus sampling can predict the presence of select CF pathogens in sputum

Patients taking ETI are much less likely to spontaneously expectorate sputum at clinic visits (42), so clinical microbiology and research laboratories are working in earnest to establish new protocols for pathogen detection, diagnosis, and monitoring in pwCF in the era of highly effective modulators. Our data set contains paired upper (sinus) and lower (sputum) airway samples, collected on the same day clinic visit, so we aimed to determine if endoscopically guided sinus sampling could serve as a replacement sampling method for patients who no longer consistently produce sputum. To evaluate concordance between microbiota in the sinus and sputum, we filtered our full data set to include only samples where we had matched upper (sinus) and lower (sputum) respiratory samples from a single visit from each subject. In total, we examined microbiota data from 108 paired samples from 21 subjects (Table 1; Fig. S2). For each genus, we sorted paired samples into one of four categories based on the presence or absence in both samples: sin+/+spu was defined by a genus being detected in both the sinus and the sputum on the same date; sin−/−spu was defined by a genus being detected in neither sample site on the same date. sin+/−spu was defined by a genus being detected in the sinus but not in the sputum on the same date; sin−/+spu was defined by a genus being detected in the sputum but not in the sinus sample from the same date.

**Fig 5:**
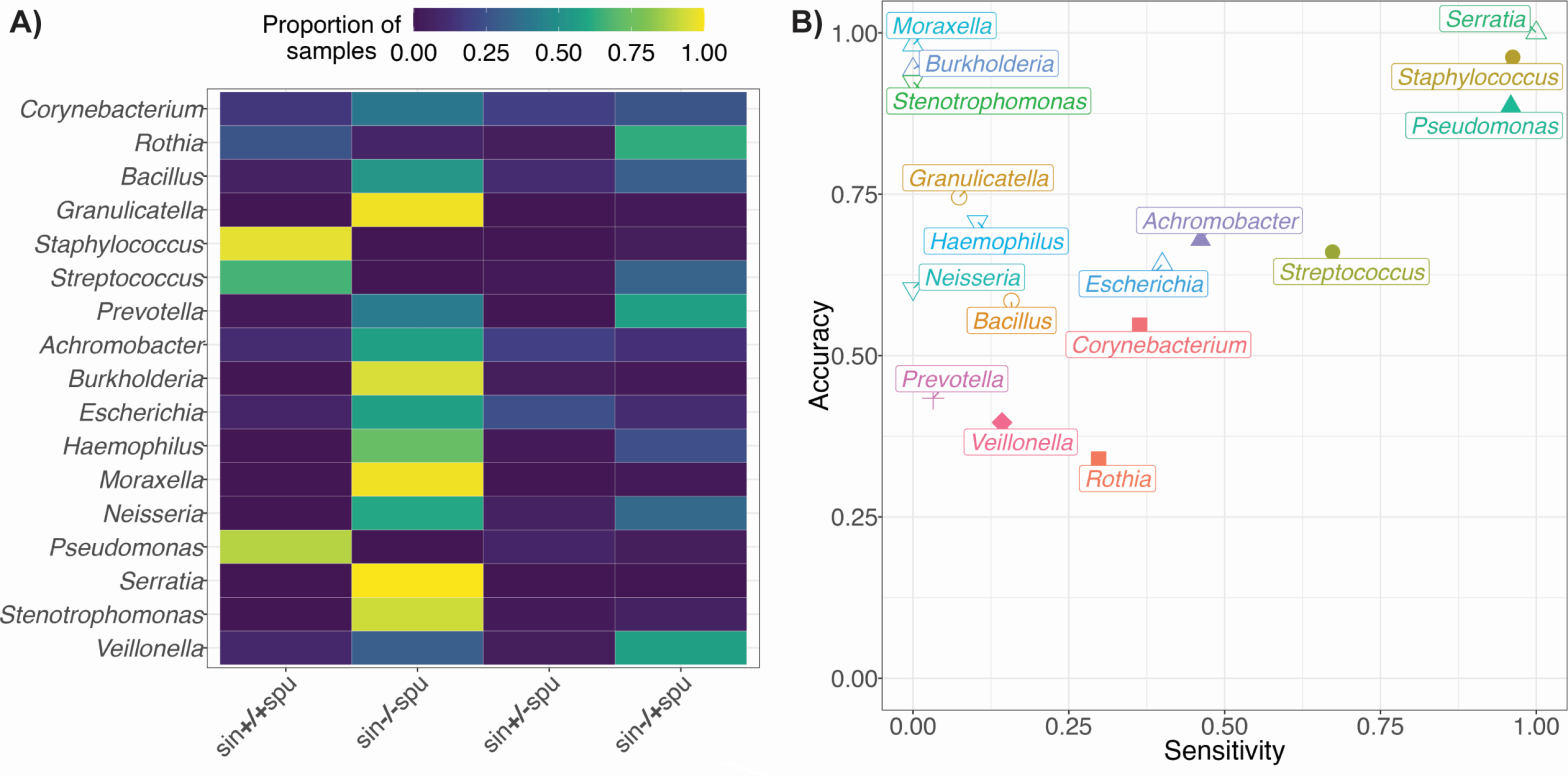
(A) Presence or absence of CF pathogens in the sputum is well predicted by sinus sampling. The color scale represents the proportion of total samples that fall into each category with dark purple indicating no samples and yellow representing all samples. (B) Microbiota concordance between the upper and lower respiratory tract is genera-dependent. Accuracy [(sin+/+spu + sin−/−spu) ÷ total] is plotted against sensitivity [sin+/+spu ÷ (sin+/+spu + sin−/+spu)]. Both accuracy and sensitivity are ratios with a range from 0 to 1 and allow quantification of a model’s ability to correctly predict outcomes. Genera with values approaching 1 for both accuracy and sensitivity have high concordance between sinus and sputum sampling.

We compared community concordance between sampling sites (Tables S2 and S3) and found presence of *Staphylococcus* spp., *Pseudomonas* spp., and *Streptococcus* spp. in the sputum was well predicted by 16S rRNA sequencing of sinus samples (Fig. 5A), although there were almost no sputum samples in this subset in which these genera were not detected. *Rothia* spp., *Prevotella* spp., and *Veillonella* spp. were more commonly detected in sputum samples when they were not detected in the paired sinus sample. By plotting accuracy against sensitivity (Table S3) we are further able to verify that sinus sampling only accurately reflects sputum microbe presence for specific genera (Fig. 5B). Genera falling in the top right quadrant of the plot (*Staphylococcus* spp., *Serratia* spp., and *Pseudomonas* spp.) are those whose presence or absence in the sputum is accurately predicted by sinus sampling. Genera present in the top left quadrant of the plot (*Moraxella* spp., *Burkholderia* spp., and *Stenotrophomonas* spp.) have their presence in the sputum well predicted by the presence in the sinus, but absence in the sinus does not accurately report an absence in the sputum. Detection of genera toward the bottom left of the plot (*Corynebacterium* spp., *Veillonella* spp., and *Prevotella* spp.) in the sputum is poorly predicted overall by sampling of the sinuses. Taken together, our results suggest that sinus sampling could be of use for monitoring prevalent CF pathogens, such as *Pseudomonas* spp. and *Staphylococcus* spp., but may not be prognostic for less commonly observed organisms.

## DISCUSSION

We have demonstrated that the sinus microbiota of pwCF is significantly altered following the initiation of ETI therapy. Individuals colonized with *Pseudomonas* experience significant reductions in the relative abundance of these taxa post-ETI, although importantly, our data show that this pathogen persists post-ETI. *Staphylococcus* spp. became the dominant bacterial organism in most patients post-ETI and we showed that this is likely due to the expansion of pathogenic MRSA in the sinuses of pwCF. We also investigated the possibility of sinus sampling as a method for monitoring CF pathogens in the lungs in the era of highly effective modulators, when many patients are no longer able to spontaneously expectorate sputum for analysis. Our results did not indicate that sinus sampling consistently predicts the taxa present in sputum samples, but is accurate for common CF pathogens and also highlights the need for increased sinus monitoring in pwCF.

It has been shown previously that the microbial community composition of the sinuses in pwCF is not stable (43) and our study also reflects this finding, with observed variation in the beta diversity of sinus samples both within and between individuals (Fig. 1B). Previous studies investigating microbial communities in the CF sinuses, including in individuals receiving surgical intervention to relieve CRS symptoms, have shown that alpha diversity in sinus samples is low and is often reduced in comparison to sputum samples from the same individuals (34, 36, 43). Subjects recruited to this study had all previously undergone FESS to relieve sinus symptoms, so the sinuses of subjects in this cohort may differ from people who have not undergone surgery. These alterations to the sinuses may include reduced temperature, increased mucus flow, and increased oxygen availability (29, 44, 45). The widened airways of people in this cohort may also allow for greater inhalation of microbes and inflammatory particulates from the environment into the sinuses (29, 46). pwCF-CRS frequently use sinonasal rinses and inhaled steroids to manage symptoms and the widened sinuses can affect penetration of these treatments into the sinuses which may lead to further differences in microbial composition in our cohort versus pwCF who have not undergone FESS (45).

The clustering of samples allowed us to identify three distinct clusters of microbiota types in the sinuses of pwCF: *Staphylococcus*-dominated samples, *Pseudomonas*-dominated samples, and samples with varied dominant organisms. The longstanding dogma in CF research is that pediatric patients frequently culture *S. aureus* throughout childhood and into their teen years before generally transitioning to becoming chronically colonized by *P. aeruginosa* (1), although recent studies contradict this and instead recognize a birth cohort effect in the Cystic Fibrosis Foundation Patient Registry data resulting from changing standards of care for CF infections (47, 48). Our cohort is from an adult population (median age at enrollment = 30 years) and we observed a greater proportion of samples dominated with *Staphylococcus* spp. than with *Pseudomonas* spp. Studies examining the microbiota of the sinuses in CF are limited, but there is evidence that the sinuses of pwCF tend to harbor a greater proportion of *Staphylococcus* spp. than the lungs (28, 33, 34) which is further supported by our study. Traditionally, *P. aeruginosa* infections in pwCF have been treated aggressively, with several studies investigating the efficacy of early eradication treatments (49–51). This treatment tactic may have led to the neglect of *S. aureus* in adult populations, which has brought about a shift in the colonization patterns of adults with CF. Although there is bidirectional movement between the upper and lower airways, they are distinct sites with differing mucosal environments and disease states, which may also play a role in the differing dynamics observed in our study and lower airway studies.

Alongside lower incidence of *Pseudomonas* spp. dominance in our sinus samples, we also observed a significant drop in *Pseudomonas* spp. relative abundance following the initiation of ETI. However, of all pwCF in this cohort with paired pre- and post-ETI samples, there was only one sample where the relative abundance of *Pseudomonas* spp. dropped to zero following ETI initiation. *Pseudomonas* spp. was detectable again in this individual’s subsequent samples. The lack of eradication of *Pseudomonas* spp. post-ETI is consistent with studies carried out using sputum samples (19, 21) and allows for the possibility of a rebound in *Pseudomonas* spp. abundance as pwCF continues to use ETI. In our samples that span several months following ETI initiation, we did not observe a rebound effect as observed in studies of the lower airway (52) leading us to believe that the sudden drop in *Pseudomonas* spp. abundance instead created an unoccupied niche leading to blooms of *Staphylococcus* spp. in the sinus, which became the dominant organism in a majority of patients. Our study samples represent less than 2 years of post-ETI data so continued surveillance will be necessary to determine if early reductions in *Pseudomonas* spp. abundance are lasting or if we will observe continued adaptation to a changing host environment and subsequent resurgence.

Commensal Staphylococci are known colonizers of the healthy nares and sinuses (53–55) so we sought to identify the species implicated in the observed bloom of Staphylococci in the sinuses post-ETI. *S. epidermidis* is present in the anterior nares of 90% of healthy individuals and *S. aureus* is an important driver of community structure in colonized individuals (53). Sinus samples in this study were obtained by endoscopically guided sheathed swabs to reduce potential contamination from the nasal microbiota during collection. Key differences between *Staphylococcus* spp. presence in healthy individuals and those with CF sinus disease are abundance and strain. Studies to characterize the healthy sinus microbiota have often been limited to 16S rRNA sequencing which means there is little information about the species-level composition of the community. However, given the prevalence of *S. epidermidis* in the nasal microbiota, it is likely that Staphylococci residing in the sinuses of healthy individuals are majority *S. epidermidis* rather than *S. aureus*, despite the prevalence of *S. aureus* colonization in healthy individuals. It is also important to consider the pathogenicity of *S. aureus* strains. MRSA is a considerable colonizer of pwCF (1) and its presence in high numbers in the sinus of pwCF is concerning. It has been demonstrated in pediatric cohorts that colonization by pathogenic bacteria is often detected first in the sinuses before ever being detected in sputum samples (56), indicating that the sinuses can act as a reservoir for bacterial movement to the lungs. The dominance of *Staphylococcus* spp. post-ETI in the sinuses of our adult cohort and the presence of MRSA raise concerns for the future of CF airway disease management in the era of HEMT. With many patients no longer able to produce sputum at clinic visits, pathogen monitoring of the lungs will become increasingly difficult or invasive. The possibility of MRSA being seeded in high numbers into the lungs from the sinuses may lead to increased numbers of chronic, antibiotic-resistant infections in the airways of pwCF going undiagnosed and untreated.

To address constraints on sampling the lungs we also investigated the ability of sinus samples to predict the microbiota of sputum samples. Our analysis showed that the predictivity of sinus sampling varied by genera. The presence of *Pseudomonas* spp. and *Staphylococcus* spp. were well predicted in the sputum microbiota by sinus sampling. This is promising for surveillance of these organisms in pwCF when sputum samples may not be readily available. An important next step in determining the utility of sinus sampling will be to investigate the relatedness of bacterial populations from the sinuses to those from the lungs through population genome sequencing. We observed that predictivity was poor for *Corynebacterium* spp. Decreases in *Corynebacterium* spp. abundance is associated with CF disease progression (54), so the lack of predictivity for this genera may be representative of the varying disease states among individuals in this subset. We found variability in the predictivity of sinus samples dependent on the genera so it is not possible to draw firm conclusions about concordance between sinus and sputum microbiota in CF. Our cohort is relatively small, so repeating this study in a large, multi-center context may provide more conclusive data that will allow clinicians to continue pathogen monitoring in the era of HEMT. This data does provide further evidence that the CF sinus should be more commonly considered when treating airway disease in pwCF and that the airway microbiota cannot be considered a monolith. Antibiotic treatment regimens for pwCF have often been decided on sputum culture alone, but we must begin to consider the effects on and disease burden of the significant bacterial community in the sinuses.

Research into the effects of HEMT on the host environment is in its infancy, but we have observed that there are significant changes to the inflammatory and nutritional profiles of the sinuses following the initiation of ETI treatment (57). Decreases in sinomucosal inflammatory cytokines were observed in individuals taking ETI, as well as decreased levels of copper, manganese, and zinc which can serve as microbial nutrients. Further work is required to mechanistically link changes in the airway environment to our observed changes in microbiota post-ETI. It is plausible that decreased inflammation and reduced nutrients available to *Pseudomonas* spp., which often becomes pathoadapted and auxotrophic in the CF airway (27), lead to the drastic reduction of *Pseudomonas* spp. abundance and allows for *Staphylococcus* spp. to become the dominant organism in the sinus post-ETI.

We acknowledge that our study has limitations. Our findings into the prevalence of *S. aureus*, particularly MRSA, are limited by the amplicon typing method used. Although we were able to generate an estimated total abundance of *Staphylococcus* spp. from 16S qPCR values, relative abundance, and 16S genome copy numbers, the technology utilized to determine strain level identification was limited to binary data (i.e., either the presence or absence of the given amplicon in each sample). Whilst we have shown that *Staphylococcus* spp. is present in high abundance in the sinuses both pre- and post-ETI and that MRSA (identified by the presence of both the *ldh1* and *mecA* and absence of *gseA* amplicons) is present in these samples, we are unable to say with certainty whether the population is a mixture of MRSA and methicillin-sensitive *S. aureus* or if MRSA is dominant. A major limitation of 16S rRNA amplicon sequencing data is that relative abundance data is compositional. This presents issues for statistical analysis when comparing alterations to microbiota between patients and over time. In the microbiome field, there have been advances in metagenomic sequencing that allow for *de novo* assembly of whole genomes from the microbial community, including bacterial, fungal, and viral members. Metagenomic sequencing also allows for greater functional analysis through the identification and classification of genes present in the sample. We were restricted to 16S rRNA amplicon sequencing of sinus samples due to low bacterial abundance and high levels of host DNA. This leads to the extraction of insufficient microbial DNA for metagenomic sequencing and genome assembly. Improved DNA extraction techniques that would allow for the depletion of host DNA could allow for metagenomic sequencing of low bacterial abundance samples in the future, but at present, these technologies do not sufficiently reduce the burden of host DNA. To overcome the limitations of relative abundance measures, future 16S rRNA sequencing would include a commercial spike-in kit that allows quantification of the absolute abundance of genera based on known quantities of rare taxa added by the user.

We have demonstrated that the sinus is an important disease site in pwCF and that pathogens residing in the sinus microbial community are not eradicated following initiation of ETI. *Pseudomonas* spp. persists in the sinus at low levels, so continued monitoring will be necessary to detect any rebound in the population. We observed blooms of *Staphylococcus* spp., including MRSA, following ETI treatment and believe that this could serve as a reservoir for future lower airway infections in pwCF, despite advances in modulator treatments. Our work and that of others show the persistence of infecting pathogens in the airway after initiation of ETI treatment, despite improved lung function and disease progression measures. Continued infection in individuals taking ETI is a concern but there also remains a population of pwCF that are not eligible for or cannot tolerate current modulator therapies, and for these individuals, it is important to continue expanding our understanding of chronic infections in CF and to drive forward developments in eradication methods.

## MATERIALS AND METHODS

### Sample collection

We performed a prospective, longitudinal study of 38 adults with CF, symptomatic CRS, and prior FESS following an IRB-approved protocol (STUDY19100149) between November 2017 and June 2021. All subjects gave informed consent. Subjects were treated in a CF-focused otolaryngology clinic at the University of Pittsburgh Medical Center. Participants were scheduled for quarterly clinic visits but also made unscheduled visits during pulmonary exacerbations. At each clinic visit a sinus swab was collected under direct endoscopic visualization from the right maxillary sinus for 16S rRNA amplicon sequencing (nylon flocked swab; Puritan Medical Products, Guildford, ME). Swabs were inserted into the sinus through a sterile sheath to ensure no contact with the nares during sampling. Expectorated sputum was collected in a sterile specimen container (Covidien general purpose specimen container; Fisher Scientific, Waltham, MA). Swabs and sputum samples were transported the same day from the clinic to the laboratory (University of Pittsburgh, Pittsburgh, PA) on wet ice before storage at −80°C.

### DNA extraction

Samples were sorted for extraction by subject and by sample type (e.g., sinus swab or sputum) and extracted in lots to reduce batch effects for intra-subject comparisons. One negative control was included per extraction lot and consisted of an unused sterile nylon flocked swab or sterile distilled water that was subjected to the complete DNA extraction protocol. DNA extraction and purification were performed using the “Benzonase 2” method described in Nelson et al. (58). An alteration was made to the hypotonic lysis stage (incubation of samples in distilled H_2_O) for sinus swabs; host cells are more readily accessible on a nylon flocked swab than in sputum and so sinus swabs were incubated at room temperature for 15 min.

### 16S rRNA and custom amplicon sequencing

Extracted and purified DNA from samples and controls was sent to DNA Services at the University of Illinois at Urbana-Champaign, IL for amplification, library preparation, and sequencing. Amplicons were generated using a microfluidics system (Standard BioTools, San Francisco, CA) which allows for multiplexed PCR of up to 48 sets of primers with 96 samples. We utilized 10 custom primer sets (Table S4) to allow strain- and species-level identification of *Pseudomonas* spp. and *Staphylococcus* spp. alongside 16S V4 (515f–806r) and ITS (ITS1f/ITS2) primers. Amplicon libraries were quantitated by qPCR and sequenced on a NovaSeq flowcell for 251 cycles from each end of the fragment using a NovaSeq 500-cycle sequencing kit v1.5 (Illumina, San Diego, CA). Generated fastq files were demultiplexed using bcl2fastq v2.20 Conversion Software (Illumina, San Diego, CA).

### 16S universal primer qPCR

Extracted and purified DNA was subjected to RT-qPCR to generate a total of 16S rRNA gene copy numbers per sample. We generated standard curves by amplifying the V4 variable region of the 16S rRNA gene using purified genomic DNA from *P*. *aeruginosa* PAO1 and the 16S V4 (515f–806r) primers (59) and quantifying total DNA produced by Qubit 4 fluorometer dsDNA broad-range assay kit (Invitrogen, San Diego, CA). 16S gene copy number was calculated and the amplified DNA product was serially diluted 1:10 and subjected in duplicate to RT-qPCR for 35 cycles under the following conditions: 94°C for 45 s, 50°C for 60 s, and 72°C for 90 s. There was a final extension at 72°C for 10 min. Mean Ct values for each dilution were plotted against Log_10_ 16S copy number and a linear regression was applied to generate a line of best fit and standard curve equation.

Sample DNA was diluted to a concentration of 1–10 ng/µL and 1 µL was used for a total reaction volume of 10 µL with iQ SYBR Green Supermix (Bio-Rad, Hercules, CA). Reactions were carried out in duplicate and mean Ct values per sample were used to calculate the 16S copy number from the standard curve equation.

The total abundance for *Staphylococcus* spp. was estimated by dividing the calculated 16S rRNA copy number by the number of 16S gene copies present in the genomes of *S. aureus* and *S. epidermidis* [each species carries five total copies of the 16S gene (60)].

### 16S rRNA amplicon analysis

All code used for analysis can be found in Supplemental data file S1. Briefly, 16S V4 amplicon data were imported into QIIME2 v2021.11 (61) using the “EMPPairedEndSequences” option. Sequences were demultiplexed using no Golay error correction and denoised using the DADA2 plug-in (62). Chimeric sequences were removed using the VSEARCH plug-in (63). Samples were rarefied to an optimal depth of 11,500 to maintain the maximum number of observed features before classifying taxa using the SILVA 138.1 rRNA database (64). Feature data were converted to a BIOM table (65) and downloaded as a tab-separated text document for further analysis in R v4.2.1.

Data were imported to phyloseq v1.40.0 (66) and amplicon sequence variants (ASVs) were decontaminated using frequency and prevalence data in samples and controls using the package decontam v1.16.0 (67). Non-bacterial taxa were filtered from the data set in phyloseq and duplicate genera were agglomerated using the function “tax_glom” and specifying genus as the taxonomic rank. CHAO1 observed Shannon and Simpson diversity indices were estimated using phyloseq. Taxa labeled “unassigned” at the genus level were extracted and their V4 variable region amplicons were extracted from the representative sequences file. Unassigned 16S sequences were queried using BLAST (68) and sequences with a >99% similarity to a given organism were manually assigned to that genus. Relative abundance per ASV per sample was calculated and these values were used for further analysis.

Beta diversity was measured by the generation of Morisita-Horn distances from relative abundance using vegan v2.6.4 (37). Hierarchical clustering was performed using the Ward clustering algorithm with the implementation of Ward’s clustering criterion and a dendrogram was generated. Package ggh4x v0.2.6 (38) was used to visualize the dendrogram as an x-axis on a taxa bar plot of samples, demonstrating the clustering of samples by dominant taxa. Per-patient centroid distances were calculated using vegan v2.6.4 (37).

We applied a logistic linear mixed effect model to pre- and post-ETI measures of Shannon diversity index and 16S copy number to determine statistically significant differences using lme4 v1.1.34 (40). Statistically significant changes in abundance and prevalence of taxa pre- and post-ETI were determined by MaAsLin 2 v1.10.0 (41) using the “LM” method with no normalization and with ETI status set as a fixed effect and patient ID set as a random effect.

### Custom amplicon analysis

Custom amplicon data were imported into QIIME2 v2021.11 (61) using the “EMPPairedEndSequences” option. Sequences were demultiplexed using no Golay error correction. Count data per primer set were converted to a BIOM table (65) and downloaded as a tab-separated text document for further analysis in R v4.2.1. The cut-off for sample positivity was set at 1% of the maximum read count per primer set to generate a binary dataframe indicating the presence or absence of given amplicons in each sample.

All code used to generate output for this manuscript can be found at https://github.com/yasminhilliam/sinus_ETI. Raw data can be found under the NCBI BioProject PRJNA1081394.

## SUPPLEMENTAL MATERIAL

Fig. S1 spectrum.00787-24-s0001.pdf Sinus samples.

Fig. S2 spectrum.00787-24-s0002.pdf Paired sinus and sputum samples

Fig. S3 spectrum.00787-24-s0003.pdf Taxa bar plot of fungal species from sinus samples.

Fig. S4 spectrum.00787-24-s0004.pdf Relative abundance plots for all genera at final pre- and all post-ETI samples.

Table S1 spectrum.00787-24-s0005.docx MaAsLin 2 analysis.

Table S2 spectrum.00787-24-s0006.docx Paired sinus and sputum sample comparisons.

Table S3 spectrum.00787-24-s0007.docx Concordance metrics per genera for paired sinus and sputum samples.

Table S4 spectrum.00787-24-s0008.docx Custom oligos used.

ASM does not own the copyrights to Supplemental Material that may be linked to, or accessed through, an article. The authors have granted ASM a non-exclusive, world-wide license to publish the Supplemental Material files. Please contact the corresponding author directly for reuse.

## References

[1] Cystic Fibrosis Foundation. 2022. Cystic Fibrosis Foundation Patient Registry Annual Data Report

[2] Mayer-Hamblett N, Rosenfeld M, Emerson J, Goss CH, Aitken ML. 2002. Developing cystic fibrosis lung transplant referral criteria using predictors of 2-year mortality. Am J Respir Crit Care Med 166:1550–1555. doi:10.1164/rccm.200202-087OC 12406843

[3] Konstan MW, Wagener JS, Vandevanter DR, Pasta DJ, Yegin A, Rasouliyan L, Morgan WJ. 2012. Risk factors for rate of decline in FEV1 in adults with cystic fibrosis. J Cyst Fibros 11:405–411. doi:10.1016/j.jcf.2012.03.009 22561369 PMC4086189

[4] Konstan MW, Morgan WJ, Butler SM, Pasta DJ, Craib ML, Silva SJ, Stokes DC, Wohl MEB, Wagener JS, Regelmann WE, Johnson CA, Scientific Advisory Group and the Investigators and Coordinators of the Epidemiologic Study of Cystic Fibrosis. 2007. Risk factors for rate of decline in forced expiratory volume in one second in children and adolescents with cystic fibrosis. J Pediatr 151:134–139, doi:10.1016/j.jpeds.2007.03.006 17643762

[5] Mayer-Hamblett Nicole, Kronmal RA, Gibson RL, Rosenfeld M, Retsch-Bogart G, Treggiari MM, Burns JL, Khan U, Ramsey BW, EPIC Investigators. 2012. Initial Pseudomonas aeruginosa treatment failure is associated with exacerbations in cystic fibrosis. Pediatr Pulmonol 47:125–134. doi:10.1002/ppul.21525 21830317 PMC3214247

[6] Dasenbrook EC, Merlo CA, Diener-West M, Lechtzin N, Boyle MP. 2008. Persistent methicillin-resistant Staphylococcus aureus and rate of FEV1 decline in cystic fibrosis. Am J Respir Crit Care Med 178:814–821. doi:10.1164/rccm.200802-327OC 18669817

[7] Vanderhelst E, De Meirleir L, Verbanck S, Piérard D, Vincken W, Malfroot A. 2012. Prevalence and impact on FEV1 decline of chronic methicillin-resistant Staphylococcus aureus (MRSA) colonization in patients with cystic fibrosis. J Cyst Fibros 11:2–7. doi:10.1016/j.jcf.2011.08.006 21907637

[8] Sanders DB, Bittner RCL, Rosenfeld M, Hoffman LR, Redding GJ, Goss CH. 2010. Failure to recover to baseline pulmonary function after cystic fibrosis pulmonary exacerbation. Am J Respir Crit Care Med 182:627–632. doi:10.1164/rccm.200909-1421OC 20463179 PMC5450763

[9] Erfanimanesh S, Emaneini M, Modaresi MR, Feizabadi MM, Halimi S, Beigverdi R, Nikbin VS, Jabalameli F. 2022. Distribution and characteristics of bacteria isolated from cystic fibrosis patients with pulmonary exacerbation. Can J Infect Dis Med Microbiol 2022:5831139. doi:10.1155/2022/5831139 36593975 PMC9805393

[10] Esposito S, Pennoni G, Mencarini V, Palladino N, Peccini L, Principi N. 2019. Antimicrobial treatment of Staphylococcus aureus in patients with cystic fibrosis. Front Pharmacol 10:849. doi:10.3389/fphar.2019.00849 31447669 PMC6692479

[11] Dicker AJ, Lonergan M, Keir HR, Smith AH, Pollock J, Finch S, Cassidy AJ, Huang JTJ, Chalmers JD. 2021. The Sputum Microbiome and clinical outcomes in patients with Bronchiectasis: a prospective observational study. Lancet Respir Med 9:885–896. doi:10.1016/S2213-2600(20)30557-9 33961805

[12] Dicker AJ, Huang JTJ, Lonergan M, Keir HR, Fong CJ, Tan B, Cassidy AJ, Finch S, Mullerova H, Miller BE, Tal-Singer R, Chalmers JD. 2021. The sputum microbiome, airway inflammation, and mortality in chronic obstructive pulmonary disease. J Allergy Clin Immunol 147:158–167. doi:10.1016/j.jaci.2020.02.040 32353489

[13] Tiew PY, Jaggi TK, Chan LLY, Chotirmall SH. 2021. The airway microbiome in COPD, bronchiectasis and bronchiectasis‐COPD overlap. Clin Respir J 15:123–133. doi:10.1111/crj.13294 33063421

[14] Loverdos K, Bellos G, Kokolatou L, Vasileiadis I, Giamarellos E, Pecchiari M, Koulouris N, Koutsoukou A, Rovina N. 2019. Lung microbiome in asthma: current perspectives. J Clin Med 8:1967. doi:10.3390/jcm8111967 31739446 PMC6912699

[15] Cuthbertson L, Walker AW, Oliver AE, Rogers GB, Rivett DW, Hampton TH, Ashare A, Elborn JS, De Soyza A, Carroll MP, Hoffman LR, Lanyon C, Moskowitz SM, O’Toole GA, Parkhill J, Planet PJ, Teneback CC, Tunney MM, Zuckerman JB, Bruce KD, van der Gast CJ. 2020. Lung function and Microbiota diversity in cystic fibrosis. Microbiome 8:45. doi:10.1186/s40168-020-00810-3 32238195 PMC7114784

[16] Bentur L, Pollak M. 2022. Trikafta—extending its success to less common mutations. J Pers Med 12:1528. doi:10.3390/jpm12091528 36143317 PMC9504046

[17] DiMango Emily, Spielman DB, Overdevest J, Keating C, Francis SF, Dansky D, Gudis DA. 2021. Effect of highly effective modulator therapy on quality of life in adults with cystic fibrosis. Int Forum Allergy Rhinol 11:75–78. doi:10.1002/alr.22700 32985756

[18] DiMango E, Overdevest J, Keating C, Francis SF, Dansky D, Gudis D. 2021. Effect of highly effective modulator treatment on sinonasal symptoms in cystic fibrosis. J Cyst Fibros 20:460–463. doi:10.1016/j.jcf.2020.07.002 32694034

[19] Nichols DP, Paynter AC, Heltshe SL, Donaldson SH, Frederick CA, Freedman SD, Gelfond D, Hoffman LR, Kelly A, Narkewicz MR, Pittman JE, Ratjen F, Rosenfeld M, Sagel SD, Schwarzenberg SJ, Singh PK, Solomon GM, Stalvey MS, Clancy JP, Kirby S, Van Dalfsen JM, Kloster MH, Rowe SM, PROMISE Study group. 2022. Clinical effectiveness of elexacaftor/tezacaftor/ivacaftor in people with cystic fibrosis: a clinical trial. Am J Respir Crit Care Med 205:529–539. doi:10.1164/rccm.202108-1986OC 34784492 PMC8906485

[20] Zhang S, Shrestha CL, Robledo-Avila F, Jaganathan D, Wisniewski BL, Brown N, Pham H, Carey K, Amer AO, Hall-Stoodley L, McCoy KS, Bai S, Partida-Sanchez S, Kopp BT. 2023. Cystic fibrosis macrophage function and clinical outcomes after elexacaftor/tezacaftor/ivacaftor. Eur Respir J 61:2102861. doi:10.1183/13993003.02861-2021 36265882 PMC10066828

[21] Durfey SL, Pipavath S, Li A, Vo AT, Ratjen A, Carter S, Morgan SJ, Radey MC, Grogan B, Salipante SJ, Welsh MJ, Stoltz DA, Goss CH, McKone EF, Singh PK. 2021. Combining Ivacaftor and intensive antibiotics achieves limited clearance of cystic fibrosis infections. mBio 12:e0314821. doi:10.1128/mbio.03148-21 34903059 PMC8669489

[22] Nichols DP, Morgan SJ, Skalland M, Vo AT, Van Dalfsen JM, Singh SB, Ni W, Hoffman LR, McGeer K, Heltshe SL, Clancy JP, Rowe SM, Jorth P, Singh PK, PROMISE-Micro Study Group. 2023. Pharmacologic improvement of CFTR function rapidly decreases Sputum pathogen density, but lung infections generally persist. J Clin Invest 133:e167957. doi:10.1172/JCI167957 36976651 PMC10178839

[23] Stevens WW, Lee RJ, Schleimer RP, Cohen NA. 2015. Chronic rhinosinusitis pathogenesis. J Allergy Clin Immunol 136:1442–1453. doi:10.1016/j.jaci.2015.10.009 26654193 PMC4680986

[24] Holm AE, Schultz HHL, Johansen HK, Pressler T, Lund TK, Iversen M, Perch M. 2021. Bacterial re-colonization occurs early after lung transplantation in cystic fibrosis patients. J Clin Med 10:1275. doi:10.3390/jcm10061275 33808547 PMC8003282

[25] Nelson J, Karempelis P, Dunitz J, Hunter R, Boyer H. 2018. Pulmonary aspiration of sinus secretions in patients with cystic fibrosis. Int Forum Allergy Rhinol 8:385–388. doi:10.1002/alr.22043 29210505

[26] Bonestroo HJC, de Winter-de Groot KM, van der Ent CK, Arets HGM. 2010. Upper and lower airway cultures in children with cystic fibrosis: do not neglect the upper Airways. J Cyst Fibros 9:130–134. doi:10.1016/j.jcf.2010.01.001 20110197

[27] Armbruster CR, Marshall CW, Garber AI, Melvin JA, Zemke AC, Moore J, Zamora PF, Li K, Fritz IL, Manko CD, Weaver ML, Gaston JR, Morris A, Methé B, DePas WH, Lee SE, Cooper VS, Bomberger JM. 2021. Adaptation and genomic erosion in fragmented Pseudomonas aeruginosa populations in the sinuses of people with cystic fibrosis. Cell Rep 37:109829. doi:10.1016/j.celrep.2021.109829 34686349 PMC8667756

[28] Zemke AC, Hilliam Y, Stapleton AL, Kimple AJ, Goralski JL, Shaffer AD, Pilewski JM, Senior BA, Lee SE, Cooper VS. 2024. Elexacaftor–tezacaftor–ivacaftor decreases pseudomonas abundance in the sinonasal microbiome in cystic fibrosis. Int Forum Allergy Rhinol 14:928–938. doi:10.1002/alr.23288 37837613 PMC11131353

[29] Chen XB, Lee HP, Chong VFH, Wang DY. 2011. Aerodynamic characteristics inside the rhino-sinonasal cavity after functional endoscopic sinus surgery. Am J Rhinol Allergy 25:388–392. doi:10.2500/ajra.2011.25.3669 22185741

[30] Giavina-Bianchi P, Aun MV, Takejima P, Kalil J, Agondi RC. 2016. United airway disease: current perspectives. J Asthma Allergy 9:93–100. doi:10.2147/JAA.S81541 27257389 PMC4872272

[31] Cho DY, Grayson JW, Woodworth BA. 2023. Unified airway—cystic fibrosis. Otolaryngol Clin North Am 56:125–136. doi:10.1016/j.otc.2022.09.009 36266104

[32] Kia’i N, Histology BT. 2023. Histology, respiratory epithelium. In Statpearls. StatPearls Publishing. https://www.ncbi.nlm.nih.gov/books/NBK541061/. 31082105

[33] Voronina OL, Ryzhova NN, Kunda MS, Loseva EV, Aksenova EI, Amelina EL, Shumkova GL, Simonova OI, Gintsburg AL. 2020. Characteristics of the airway Microbiome of cystic fibrosis patients. Biochemistry (Mosc) 85:1–10. doi:10.1134/S0006297920010010 32079513

[34] Lucas SK, Yang R, Dunitz JM, Boyer HC, Hunter RC. 2018. 16S rRNA gene sequencing reveals site-specific signatures of the upper and lower airways of cystic fibrosis patients. J Cyst Fibros 17:204–212. doi:10.1016/j.jcf.2017.08.007 28826586 PMC5817045

[35] Paramasivan S, Bassiouni A, Shiffer A, Dillon MR, Cope EK, Cooksley C, Ramezanpour M, Moraitis S, Ali MJ, Bleier B, Callejas C, Cornet ME, Douglas RG, Dutra D, Georgalas C, Harvey RJ, Hwang PH, Luong AU, Schlosser RJ, Tantilipikorn P, Tewfik MA, Vreugde S, Wormald P-J, Caporaso JG, Psaltis AJ. 2020. The international sinonasal microbiome study: a multicentre, multinational characterization of sinonasal bacterial ecology. Allergy 75:2037–2049. doi:10.1111/all.14276 32167574

[36] Cope EK, Goldberg AN, Pletcher SD, Lynch SV. 2017. Compositionally and functionally distinct sinus microbiota in chronic rhinosinusitis patients have immunological and clinically divergent consequences. Microbiome 5:53. doi:10.1186/s40168-017-0266-6 28494786 PMC5427582

[37] Oksanen J, Simpson G, Blanchet F, Kindt R, Legendre P, Minchin P, et al.. 2022. R package vegan version 2.6-4. Community ecology package. Available from: https://CRAN.R-project.org/package=vegan. Retrieved 26 Nov 2023.

[38] T van den B. 2023. R package version 0.2.6. Ggh4x: hacks for “ggplot2. Available from: https://CRAN.R-project.org/package=ggh4x. Retrieved 26 Nov 2023.

[39] Kuznetsova A, Brockhoff PB, Christensen RHB. 2017. lmerTest package: tests in linear mixed effects models. J Stat Soft 82. doi:10.18637/jss.v082.i13

[40] Bates D, Mächler M, Bolker B, Walker S. 2015. Fitting linear mixed-effects models using Lme4. J Stat Softw 67. doi:10.18637/jss.v067.i01

[41] Mallick H, Rahnavard A, McIver L. 2020. R/Bioconductor package. MaAsLin 2: multivariable association in population-scale meta-Omics studies. Available from: http://huttenhower.sph.harvard.edu/maaslin2. Retrieved 26 Nov 2023.

[42] Hisert KB, Birket SE, Clancy JP, Downey DG, Engelhardt JF, Fajac I, Gray RD, Lachowicz-Scroggins ME, Mayer-Hamblett N, Thibodeau P, Tuggle KL, Wainwright CE, De Boeck K. 2023. Understanding and addressing the needs of people with cystic fibrosis in the era of CFTR modulator therapy. Lancet Respir Med 11:916–931. doi:10.1016/S2213-2600(23)00324-7 37699420 PMC13005650

[43] Armbruster CR, Li K, Kiedrowski MR, Zemke AC, Melvin JA, Moore J, Atteih S, Fitch AC, DuPont M, Manko CD, Weaver ML, Gaston JR, Alcorn JF, Morris A, Methé BA, Lee SE, Bomberger JM. 2022. Low diversity and instability of the sinus microbiota over time in adults with cystic fibrosis. Microbiol Spectr 10:e0125122. doi:10.1128/spectrum.01251-22 36094193 PMC9603634

[44] Naraghi M, Baghbanian N, Moharari M, Saghazadeh A. 2018. Improvement of sinonasal mucociliary function by endoscopic sinus surgery in patients with chronic rhinosinusitis. Am J Otolaryngol 39:707–710. doi:10.1016/j.amjoto.2018.07.019 30131169

[45] Jain R, Kumar H, Tawhai M, Douglas R. 2017. The impact of endoscopic sinus surgery on paranasal physiology in simulated sinus cavities. Int Forum Allergy Rhinol 7:248–255. doi:10.1002/alr.21879 27869357

[46] Arias-Pérez RD, Taborda NA, Gómez DM, Narvaez JF, Porras J, Hernandez JC. 2020. Inflammatory effects of particulate matter air pollution. Environ Sci Pollut Res Int 27:42390–42404. doi:10.1007/s11356-020-10574-w 32870429

[47] VanDevanter DR, LiPuma JJ, Konstan MW. 2024. Longitudinal bacterial prevalence in cystic fibrosis airways: fact and Artifact. J Cyst Fibros 23:58–64. doi:10.1016/j.jcf.2023.09.011 37783605 PMC10949087

[48] Fischer AJ, Planet PJ. 2024. A birth cohort approach to understanding cystic fibrosis lung infections. J Cyst Fibros 23:8–11. doi:10.1016/j.jcf.2023.10.014 37949746

[49] Hansen CR, Pressler T, Høiby N. 2008. Early aggressive eradication therapy for intermittent Pseudomonas aeruginosa airway colonization in cystic fibrosis patients: 15 years experience. J Cyst Fibros 7:523–530. doi:10.1016/j.jcf.2008.06.009 18693078

[50] Treggiari MM, Rosenfeld M, Retsch-Bogart G, Gibson R, Ramsey B. 2007. Approach to eradication of initial Pseudomonas aeruginosa infection in children with cystic fibrosis. Pediatr Pulmonol 42:751–756. doi:10.1002/ppul.20665 17647287

[51] Mayer-Hamblett N, Kloster M, Rosenfeld M, Gibson RL, Retsch-Bogart GZ, Emerson J, Thompson V, Ramsey BW. 2015. Impact of sustained eradication of new Pseudomonas aeruginosa infection on long-term outcomes in cystic fibrosis. Clin Infect Dis 61:707–715. doi:10.1093/cid/civ377 25972024 PMC4626753

[52] Hisert KB, Heltshe SL, Pope C, Jorth P, Wu X, Edwards RM, Radey M, Accurso FJ, Wolter DJ, Cooke G, Adam RJ, Carter S, Grogan B, Launspach JL, Donnelly SC, Gallagher CG, Bruce JE, Stoltz DA, Welsh MJ, Hoffman LR, McKone EF, Singh PK. 2017. Restoring cystic fibrosis transmembrane conductance regulator function reduces airway bacteria and inflammation in people with cystic fibrosis and chronic lung infections. Am J Respir Crit Care Med 195:1617–1628. doi:10.1164/rccm.201609-1954OC 28222269 PMC5476912

[53] Liu CM, Price LB, Hungate BA, Abraham AG, Larsen LA, Christensen K, Stegger M, Skov R, Andersen PS. 2015. Staphylococcus aureus and the ecology of the nasal microbiome. Sci Adv 1:e1400216. doi:10.1126/sciadv.1400216 26601194 PMC4640600

[54] Hoggard M, Biswas K, Zoing M, Wagner Mackenzie B, Taylor MW, Douglas RG. 2017. Evidence of microbiota dysbiosis in chronic rhinosinusitis. Int Forum Allergy Rhinol 7:230–239. doi:10.1002/alr.21871 27879060

[55] Lal D, Keim P, Delisle J, Barker B, Rank MA, Chia N, Schupp JM, Gillece JD, Cope EK. 2017. Mapping and comparing bacterial microbiota in the sinonasal cavity of healthy, allergic rhinitis, and chronic rhinosinusitis subjects. Int Forum Allergy Rhinol 7:561–569. doi:10.1002/alr.21934 28481057

[56] Bonestroo HJC, de Winter-de Groot KM, van der Ent CK, Arets HGM. 2010. Upper and lower airway cultures in children with cystic fibrosis: do not neglect the upper airways. J Cyst Fibros 9:130–134. doi:10.1016/j.jcf.2010.01.001 20110197

[57] Atteih SE, Armbruster CR, Hilliam Y, Rapsinski GJ, Bhusal JK, Krainz LL, Gaston JR, DuPont M, Zemke AC, Alcorn JF, Moore JA, Cooper VS, Lee SE, Forno E, Bomberger JM. 2024. Effects of highly effective modulator therapy on the dynamics of the respiratory mucosal environment in cystic fibrosis. Pediatr Pulmonol 59:1266–1273. doi:10.1002/ppul.26898 38353361 PMC11058019

[58] Nelson MT, Pope CE, Marsh RL, Wolter DJ, Weiss EJ, Hager KR, Vo AT, Brittnacher MJ, Radey MC, Hayden HS, Eng A, Miller SI, Borenstein E, Hoffman LR. 2019. Human and extracellular DNA depletion for metagenomic analysis of complex clinical infection samples yields optimized viable microbiome profiles. Cell Reports 26:2227–2240. doi:10.1016/j.celrep.2019.01.091 30784601 PMC6435281

[59] Walters W, Hyde ER, Berg-Lyons D, Ackermann G, Humphrey G, Parada A, Gilbert JA, Jansson JK, Caporaso JG, Fuhrman JA, Apprill A, Knight R. 2016. Improved bacterial 16S rRNA gene (V4 and V4-5) and fungal internal transcribed spacer marker gene primers for microbial community surveys. mSystems 1. doi:10.1128/mSystems.00009-15 PMC5069754 27822518

[60] Winand R, Bogaerts B, Hoffman S, Lefevre L, Delvoye M, Braekel JV, Fu Q, Roosens NH, Keersmaecker SCD, Vanneste K. 2019. Targeting the 16S rRNA gene for bacterial identification in complex mixed samples: comparative evaluation of second (Illumina) and third (oxford nanopore technologies) generation sequencing technologies. Int J Mol Sci 21:298. doi:10.3390/ijms21010298 31906254 PMC6982111

[61] Bolyen E, Rideout JR, Dillon MR, Bokulich NA, Abnet CC, Al-Ghalith GA, Alexander H, Alm EJ, Arumugam M, Asnicar F, et al.. 2019. Reproducible, interactive, scalable and extensible microbiome data science using QIIME 2. Nat Biotechnol 37:852–857. doi:10.1038/s41587-019-0209-9 31341288 PMC7015180

[62] Callahan BJ, McMurdie PJ, Rosen MJ, Han AW, Johnson AJA, Holmes SP. 2016. DADA2: high-resolution sample inference from Illumina Amplicon data. Nat Methods 13:581–583. doi:10.1038/nmeth.3869 27214047 PMC4927377

[63] Rognes T, Flouri T, Nichols B, Quince C, Mahé F. 2016. VSEARCH: a versatile open source tool for metagenomics. PeerJ 4:e2584. doi:10.7717/peerj.2584 27781170 PMC5075697

[64] Quast C, Pruesse E, Yilmaz P, Gerken J, Schweer T, Yarza P, Peplies J, Glöckner FO. 2013. The SILVA ribosomal RNA gene database project: improved data processing and web-based tools. Nucleic Acids Res 41:D590–6. doi:10.1093/nar/gks1219 23193283 PMC3531112

[65] McDonald D, Clemente JC, Kuczynski J, Rideout JR, Stombaugh J, Wendel D, Wilke A, Huse S, Hufnagle J, Meyer F, Knight R, Caporaso JG. 2012. The biological observation matrix (BIOM) format or: how I learned to stop worrying and love the OME-OME. Gigascience 1:7. doi:10.1186/2047-217X-1-7 23587224 PMC3626512

[66] McMurdie PJ, Holmes S. 2013. An R package for reproducible interactive analysis and graphics of microbiome census data. PLoS One 8:e61217. doi:10.1371/journal.pone.0061217 23630581 PMC3632530

[67] Davis NM, Proctor DM, Holmes SP, Relman DA, Callahan BJ. 2018. Simple statistical identification and removal of contaminant sequences in marker-gene and metagenomics data. Microbiome 6:226. doi:10.1186/s40168-018-0605-2 30558668 PMC6298009

[68] Altschul SF, Gish W, Miller W, Myers EW, Lipman DJ. 1990. Basic local alignment search tool. J Mol Biol 215:403–410. doi:10.1016/S0022-2836(05)80360-2 2231712

