# Supplementary material for: Cystic fibrosis pathogens persist in the upper respiratory tract following initiation of elexacaftor/tezacaftor/ivacaftor therapy
Source: Microbiol Spectr. 2024 Jun 25;12(8):e00787-24. doi: 10.1128/spectrum.00787-24 (PMC11302335; doi:10.1128/spectrum.00787-24)
Supplement: Fig. S1 — Sinus samples. [file spectrum.00787-24-s0001.pdf]

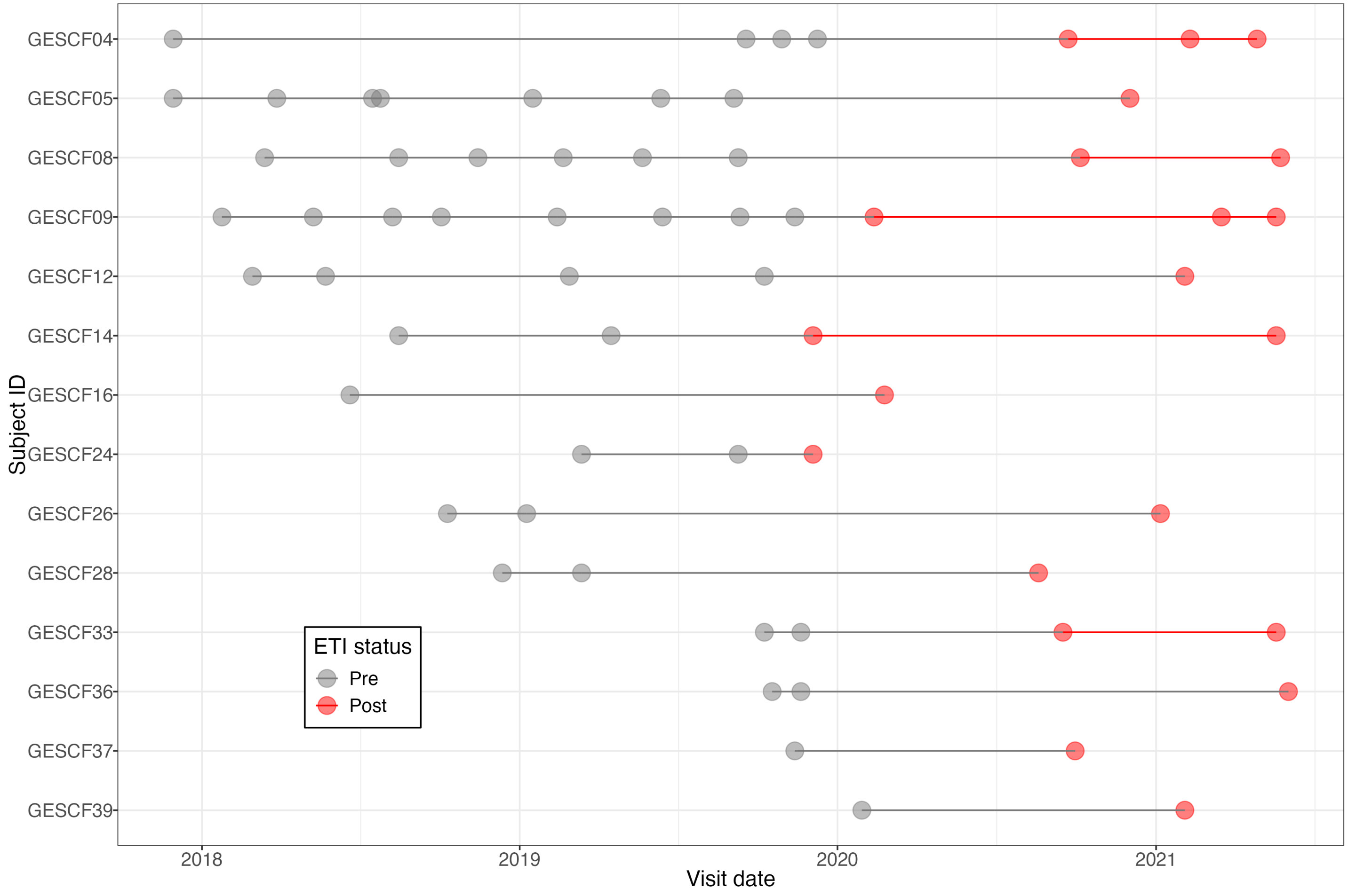

Figure S1: Sinus sample collection dates per subject for 16S rRNA amplicon sequencing. Grey points indicate pre-ETI samples; red points indicate post-ETI samples.
