# Supplementary material for: Cystic fibrosis pathogens persist in the upper respiratory tract following initiation of elexacaftor/tezacaftor/ivacaftor therapy
Source: Microbiol Spectr. 2024 Jun 25;12(8):e00787-24. doi: 10.1128/spectrum.00787-24 (PMC11302335; doi:10.1128/spectrum.00787-24)
Supplement: Fig. S2 — Paired sinus and sputum samples [file spectrum.00787-24-s0002.pdf]

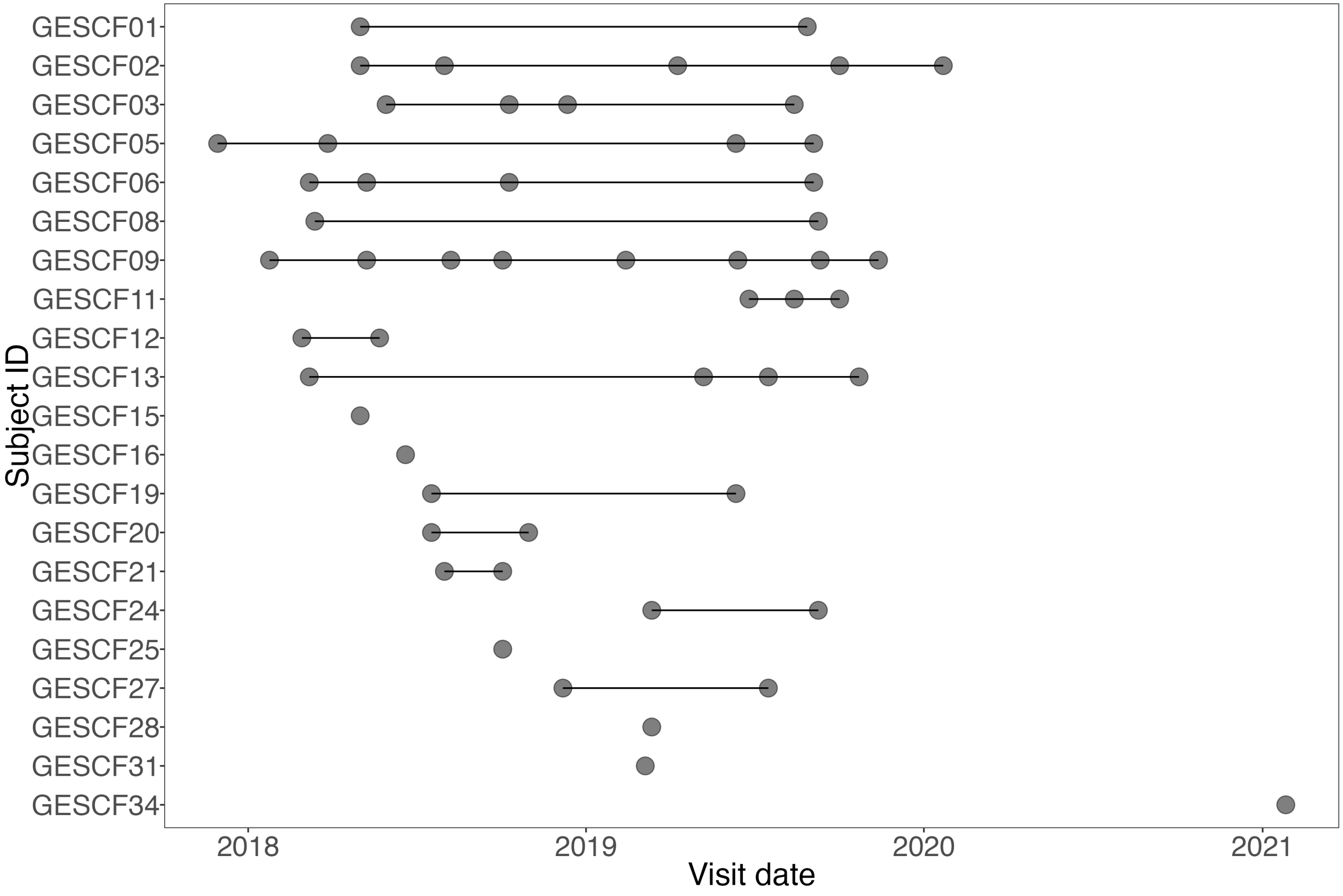

Figure S2: Paired sinus and sputum sample collection dates per subject. Each point represents a date on which both a sinus and sputum sample were collected from the same subject.
