# Supplementary material for: Cystic fibrosis pathogens persist in the upper respiratory tract following initiation of elexacaftor/tezacaftor/ivacaftor therapy
Source: Microbiol Spectr. 2024 Jun 25;12(8):e00787-24. doi: 10.1128/spectrum.00787-24 (PMC11302335; doi:10.1128/spectrum.00787-24)
Supplement: Fig. S3 — Taxa bar plot of fungal species from sinus samples. [file spectrum.00787-24-s0003.pdf]

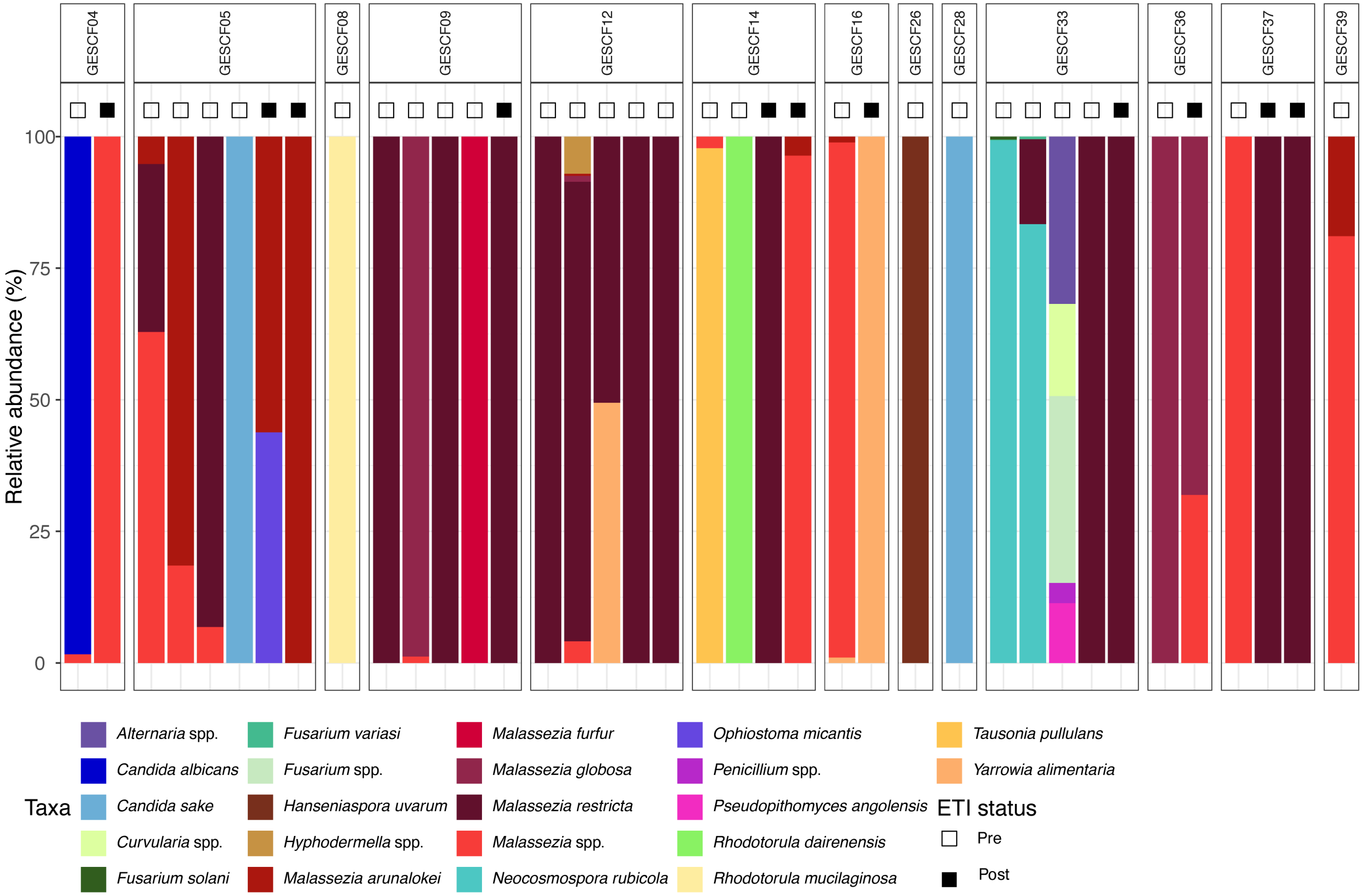

Figure S3: Relative abundance of fungal taxa identified by ITS amplicon sequencing and the ETI status of the subject at time of sample collection.
