# Supplementary material for: Cystic fibrosis pathogens persist in the upper respiratory tract following initiation of elexacaftor/tezacaftor/ivacaftor therapy
Source: Microbiol Spectr. 2024 Jun 25;12(8):e00787-24. doi: 10.1128/spectrum.00787-24 (PMC11302335; doi:10.1128/spectrum.00787-24)
Supplement: Fig. S4 — Relative abundance plots for all genera at final pre- and all post-ETI samples. [file spectrum.00787-24-s0004.pdf]

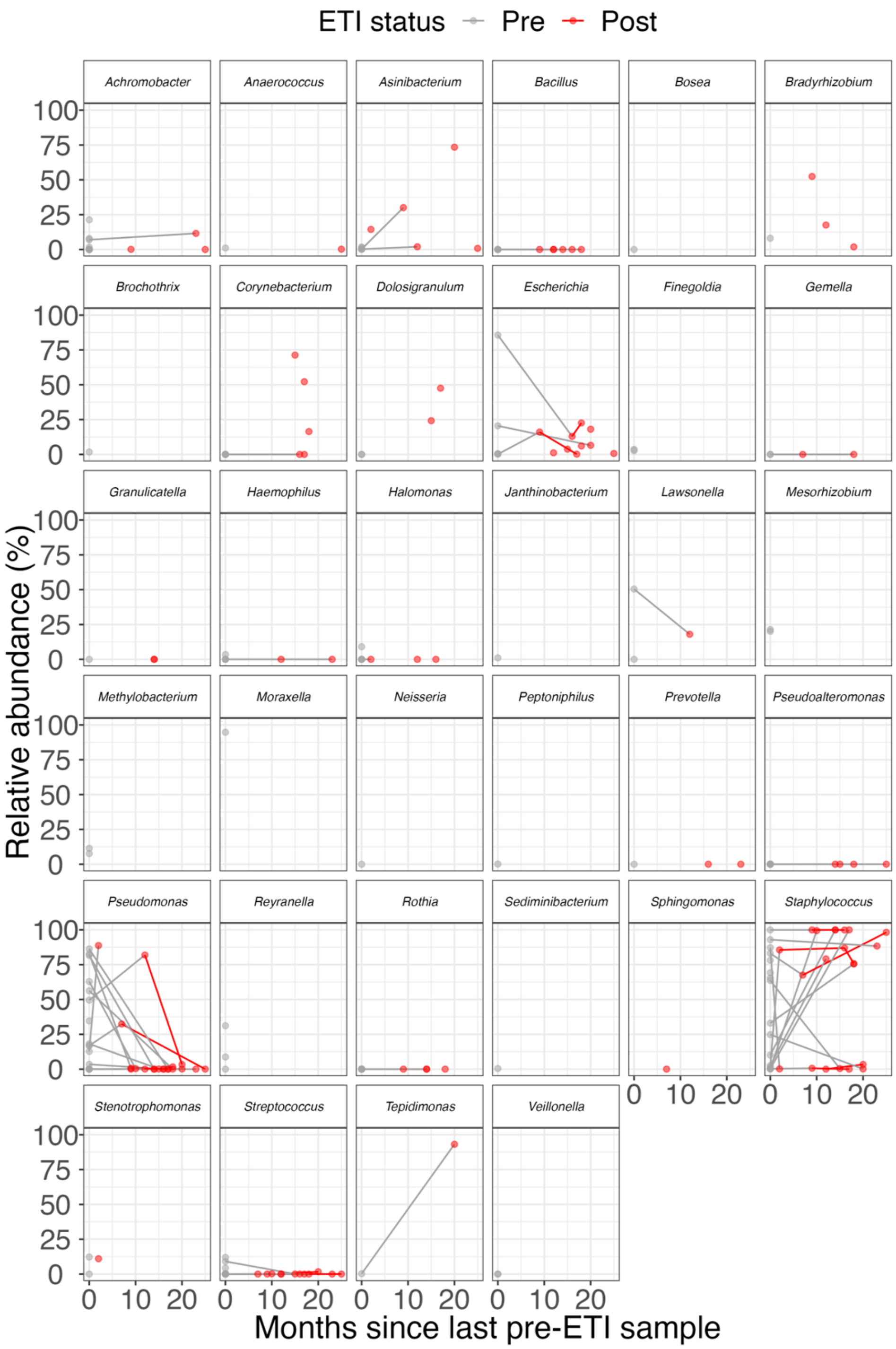

Figure S4: Relative abundance at last available pre-ETI sample and all post-ETI samples for all genera and patients.
