# Supplementary material for: Cystic fibrosis pathogens persist in the upper respiratory tract following initiation of elexacaftor/tezacaftor/ivacaftor therapy
Source: Microbiol Spectr. 2024 Jun 25;12(8):e00787-24. doi: 10.1128/spectrum.00787-24 (PMC11302335; doi:10.1128/spectrum.00787-24)
Supplement: Table S1 — MaAsLin 2 analysis. [file spectrum.00787-24-s0005.docx]

Table S1: Full data table from MaAsLin 2 analysis arranged by ascending *P* value.

| **feature** | **metadata** | **value** | **coef** | **stderr** | **N** | **N.not.0** | **pval** | **qval** |
| --- | --- | --- | --- | --- | --- | --- | --- | --- |
| *Pseudomonas* | ETI | post | -4.8115975 | 1.09789223 | 65 | 62 | 5.46164408895336e-05 | 0.00240312 |
| *Asinibacterium* | ETI | post | 1.24354811 | 0.47006114 | 65 | 9 | 0.01028687 | 0.22631112 |
| *Moraxella* | ETI | post | -0.5855768 | 0.39912321 | 65 | 3 | 0.14903791 | 0.60818347 |
| *Achromobacter* | ETI | post | -1.1068782 | 0.60487527 | 65 | 17 | 0.0732106 | 0.60818347 |
| *Lawsonella* | ETI | post | -0.702889 | 0.45735965 | 65 | 8 | 0.13043017 | 0.60818347 |
| *Dolosigranulum* | ETI | post | 1.20595748 | 0.57883692 | 65 | 3 | 0.04173158 | 0.60818347 |
| *Streptococcus* | ETI | post | -1.025373 | 0.72508795 | 65 | 45 | 0.16276866 | 0.60818347 |
| *Mesorhizobium* | ETI | post | -0.0340239 | 0.0273431 | 65 | 1 | 0.21958641 | 0.60818347 |
| *Bradyrhizobium* | ETI | post | 0.35533163 | 0.23728278 | 65 | 5 | 0.14064849 | 0.60818347 |
| *Escherichia* | ETI | post | 1.33566167 | 0.696527 | 65 | 21 | 0.06046674 | 0.60818347 |
| *Methylobacterium* | ETI | post | -0.559881 | 0.39451379 | 65 | 3 | 0.16248597 | 0.60818347 |
| *Finegoldia* | ETI | post | -0.5327986 | 0.30683546 | 65 | 8 | 0.08790066 | 0.60818347 |
| *Brochothrix* | ETI | post | -0.0340239 | 0.0273431 | 65 | 1 | 0.21958641 | 0.60818347 |
| *Janthinobacterium* | ETI | post | -0.0340239 | 0.0273431 | 65 | 1 | 0.21958641 | 0.60818347 |
| *Sediminibacterium* | ETI | post | -0.0340239 | 0.0273431 | 65 | 1 | 0.21958641 | 0.60818347 |
| *Neisseria* | ETI | post | -0.0959975 | 0.07743904 | 65 | 3 | 0.22115762 | 0.60818347 |
| *Staphylococcus* | ETI | post | 0.7423937 | 0.80731082 | 65 | 64 | 0.36194745 | 0.62100052 |
| *Corynebacterium* | ETI | post | 0.7751409 | 0.89579764 | 65 | 19 | 0.39047714 | 0.62100052 |
| *Actinomyces* | ETI | post | -0.0261473 | 0.03246668 | 65 | 1 | 0.42415432 | 0.62100052 |
| *Eikenella* | ETI | post | -0.0229441 | 0.03300686 | 65 | 1 | 0.48964399 | 0.62100052 |
| *Reyranella* | ETI | post | -0.5913242 | 0.55039037 | 65 | 4 | 0.28675359 | 0.62100052 |
| *Haemophilus* | ETI | post | -0.5162878 | 0.45496558 | 65 | 9 | 0.2612758 | 0.62100052 |
| *Acinetobacter* | ETI | post | -0.0227273 | 0.03303378 | 65 | 1 | 0.49397769 | 0.62100052 |
| *Lachnoanaerobaculum* | ETI | post | -0.0261473 | 0.03246668 | 65 | 1 | 0.42415432 | 0.62100052 |
| *Rhodococcus* | ETI | post | -0.0227273 | 0.03303378 | 65 | 1 | 0.49397769 | 0.62100052 |
| *Peptoniphilus* | ETI | post | -0.3199566 | 0.28754184 | 65 | 4 | 0.27053332 | 0.62100052 |
| Unassigned | ETI | post | -0.0261473 | 0.03246668 | 65 | 1 | 0.42415432 | 0.62100052 |
| *Campylobacter* | ETI | post | -0.0229441 | 0.03300686 | 65 | 1 | 0.48964399 | 0.62100052 |
| *Veillonella* | ETI | post | -0.2240958 | 0.19483084 | 65 | 3 | 0.25516323 | 0.62100052 |
| *Phreatobacter* | ETI | post | -0.0245886 | 0.03164484 | 65 | 1 | 0.4409369 | 0.62100052 |
| *Rothia* | ETI | post | -0.1844142 | 0.18196224 | 65 | 14 | 0.31470983 | 0.62100052 |
| *Halomonas* | ETI | post | -0.311679 | 0.38220987 | 65 | 10 | 0.41787817 | 0.62100052 |
| *Pandoraea* | ETI | post | -0.0227273 | 0.03303378 | 65 | 1 | 0.49397769 | 0.62100052 |
| *Burkholderia* | ETI | post | -0.0245886 | 0.03164484 | 65 | 1 | 0.4409369 | 0.62100052 |
| *Idiomarina* | ETI | post | -0.0744213 | 0.08137132 | 65 | 2 | 0.36389478 | 0.62100052 |
| *Granulicatella* | ETI | post | -0.2706239 | 0.41728248 | 65 | 5 | 0.51937336 | 0.63478967 |
| *Tepidimonas* | ETI | post | 0.18353842 | 0.32251354 | 65 | 3 | 0.57199455 | 0.66991053 |
| *Pseudoalteromonas* | ETI | post | -0.1733733 | 0.31032927 | 65 | 15 | 0.57855909 | 0.66991053 |
| *Sphingomonas* | ETI | post | -0.0693183 | 0.17306689 | 65 | 2 | 0.69012216 | 0.77859937 |
| *Stenotrophomonas* | ETI | post | -0.0937015 | 0.49127931 | 65 | 4 | 0.84947813 | 0.87736285 |
| *Anaerococcus* | ETI | post | -0.0256331 | 0.11135309 | 65 | 2 | 0.81868487 | 0.87736285 |
| *Prevotella* | ETI | post | -0.0419824 | 0.23265655 | 65 | 4 | 0.85742278 | 0.87736285 |
| *Gemella* | ETI | post | 0.01569589 | 0.06820943 | 65 | 5 | 0.81895064 | 0.87736285 |
| *Bacillus* | ETI | post | 0.03187672 | 0.25727376 | 65 | 16 | 0.90178739 | 0.90178739 |
