# Supplementary material for: Cystic fibrosis pathogens persist in the upper respiratory tract following initiation of elexacaftor/tezacaftor/ivacaftor therapy
Source: Microbiol Spectr. 2024 Jun 25;12(8):e00787-24. doi: 10.1128/spectrum.00787-24 (PMC11302335; doi:10.1128/spectrum.00787-24)
Supplement: Table S2 — Paired sinus and sputum sample comparisons. [file spectrum.00787-24-s0006.docx]

Table S2: Full table of proportion of samples in each category per genera for sinus and sputum microbiome comparisons.

| **genus** | **category** | **proportion** |
| --- | --- | --- |
| *Staphylococcus* | sin+/+spu | 0.96226415 |
| *Staphylococcus* | sin-/-spu | 0 |
| *Staphylococcus* | sin+/-spu | 0 |
| *Staphylococcus* | sin-/+spu | 0.03773585 |
| *Pseudomonas* | sin+/+spu | 0.88679245 |
| *Pseudomonas* | sin-/-spu | 0 |
| *Pseudomonas* | sin+/-spu | 0.0754717 |
| *Pseudomonas* | sin-/+spu | 0.03773585 |
| *Burkholderia* | sin+/+spu | 0 |
| *Burkholderia* | sin-/-spu | 0.94339623 |
| *Burkholderia* | sin+/-spu | 0.03773585 |
| *Burkholderia* | sin-/+spu | 0.01886792 |
| *Achromobacter* | sin+/+spu | 0.11320755 |
| *Achromobacter* | sin-/-spu | 0.56603774 |
| *Achromobacter* | sin+/-spu | 0.18867925 |
| *Achromobacter* | sin-/+spu | 0.13207547 |
| *Rothia* | sin+/+spu | 0.26415094 |
| *Rothia* | sin-/-spu | 0.0754717 |
| *Rothia* | sin+/-spu | 0.03773585 |
| *Rothia* | sin-/+spu | 0.62264151 |
| *Lawsonella* | sin+/+spu | 0.01886792 |
| *Lawsonella* | sin-/-spu | 0.8490566 |
| *Lawsonella* | sin+/-spu | 0.0754717 |
| *Lawsonella* | sin-/+spu | 0.05660377 |
| *Streptococcus* | sin+/+spu | 0.66037736 |
| *Streptococcus* | sin-/-spu | 0 |
| *Streptococcus* | sin+/-spu | 0.01886792 |
| *Streptococcus* | sin-/+spu | 0.32075472 |
| *Fusobacterium* | sin+/+spu | 0 |
| *Fusobacterium* | sin-/-spu | 0.64150943 |
| *Fusobacterium* | sin+/-spu | 0 |
| *Fusobacterium* | sin-/+spu | 0.35849057 |
| *Haemophilus* | sin+/+spu | 0.05660377 |
| *Haemophilus* | sin-/-spu | 0.62264151 |
| *Haemophilus* | sin+/-spu | 0.0754717 |
| *Haemophilus* | sin-/+spu | 0.24528302 |
| *Stenotrophomonas* | sin+/+spu | 0 |
| *Stenotrophomonas* | sin-/-spu | 0.9245283 |
| *Stenotrophomonas* | sin+/-spu | 0.01886792 |
| *Stenotrophomonas* | sin-/+spu | 0.05660377 |
| *Actinomyces* | sin+/+spu | 0.01886792 |
| *Actinomyces* | sin-/-spu | 0.16981132 |
| *Actinomyces* | sin+/-spu | 0 |
| *Actinomyces* | sin-/+spu | 0.81132075 |
| *Veillonella* | sin+/+spu | 0.09433962 |
| *Veillonella* | sin-/-spu | 0.30188679 |
| *Veillonella* | sin+/-spu | 0.03773585 |
| *Veillonella* | sin-/+spu | 0.56603774 |
| *Pandoraea* | sin+/+spu | 0 |
| *Pandoraea* | sin-/-spu | 0.9245283 |
| *Pandoraea* | sin+/-spu | 0.01886792 |
| *Pandoraea* | sin-/+spu | 0.05660377 |
| *Haemophilus* | sin+/+spu | 0 |
| *Haemophilus* | sin-/-spu | 0.73584906 |
| *Haemophilus* | sin+/-spu | 0.01886792 |
| *Haemophilus* | sin-/+spu | 0.24528302 |
| *Neisseria* | sin+/+spu | 0 |
| *Neisseria* | sin-/-spu | 0.60377358 |
| *Neisseria* | sin+/-spu | 0.05660377 |
| *Neisseria* | sin-/+spu | 0.33962264 |
| *Methylobacterium* | sin+/+spu | 0 |
| *Methylobacterium* | sin-/-spu | 0.94339623 |
| *Methylobacterium* | sin+/-spu | 0.03773585 |
| *Methylobacterium* | sin-/+spu | 0.01886792 |
| *Enterococcus* | sin+/+spu | 0 |
| *Enterococcus* | sin-/-spu | 0.94339623 |
| *Enterococcus* | sin+/-spu | 0 |
| *Enterococcus* | sin-/+spu | 0.05660377 |
| *Gemella* | sin+/+spu | 0.03773585 |
| *Gemella* | sin-/-spu | 0.32075472 |
| *Gemella* | sin+/-spu | 0 |
| *Gemella* | sin-/+spu | 0.64150943 |
| *Corynebacterium* | sin+/+spu | 0.1509434 |
| *Corynebacterium* | sin-/-spu | 0.39622642 |
| *Corynebacterium* | sin+/-spu | 0.18867925 |
| *Corynebacterium* | sin-/+spu | 0.26415094 |
| *Porphyromonas* | sin+/+spu | 0 |
| *Porphyromonas* | sin-/-spu | 0.73584906 |
| *Porphyromonas* | sin+/-spu | 0 |
| *Porphyromonas* | sin-/+spu | 0.26415094 |
| *Prevotella* | sin+/+spu | 0.01886792 |
| *Prevotella* | sin-/-spu | 0.41509434 |
| *Prevotella* | sin+/-spu | 0 |
| *Prevotella* | sin-/+spu | 0.56603774 |
| *Mesorhizobium* | sin+/+spu | 0 |
| *Mesorhizobium* | sin-/-spu | 0.98113208 |
| *Mesorhizobium* | sin+/-spu | 0.01886792 |
| *Mesorhizobium* | sin-/+spu | 0 |
| *Granulicatella* | sin+/+spu | 0.03773585 |
| *Granulicatella* | sin-/-spu | 0.47169811 |
| *Granulicatella* | sin+/-spu | 0.03773585 |
| *Granulicatella* | sin-/+spu | 0.45283019 |
| *Lachnoanaerobaculum* | sin+/+spu | 0 |
| *Lachnoanaerobaculum* | sin-/-spu | 0.71698113 |
| *Lachnoanaerobaculum* | sin+/-spu | 0 |
| *Lachnoanaerobaculum* | sin-/+spu | 0.28301887 |
| *Reyranella* | sin+/+spu | 0 |
| *Reyranella* | sin-/-spu | 0.90566038 |
| *Reyranella* | sin+/-spu | 0.0754717 |
| *Reyranella* | sin-/+spu | 0.01886792 |
| *Escherichia* | sin+/+spu | 0.0754717 |
| *Escherichia* | sin-/-spu | 0.56603774 |
| *Escherichia* | sin+/-spu | 0.24528302 |
| *Escherichia* | sin-/+spu | 0.11320755 |
| *Solobacterium* | sin+/+spu | 0 |
| *Solobacterium* | sin-/-spu | 0.62264151 |
| *Solobacterium* | sin+/-spu | 0.01886792 |
| *Solobacterium* | sin-/+spu | 0.35849057 |
| *Leptotrichia* | sin+/+spu | 0 |
| *Leptotrichia* | sin-/-spu | 0.81132075 |
| *Leptotrichia* | sin+/-spu | 0 |
| *Leptotrichia* | sin-/+spu | 0.18867925 |
| *Alloprevotella* | sin+/+spu | 0 |
| *Alloprevotella* | sin-/-spu | 0.81132075 |
| *Alloprevotella* | sin+/-spu | 0 |
| *Alloprevotella* | sin-/+spu | 0.18867925 |
| *Finegoldia* | sin+/+spu | 0 |
| *Finegoldia* | sin-/-spu | 0.83018868 |
| *Finegoldia* | sin+/-spu | 0.09433962 |
| *Finegoldia* | sin-/+spu | 0.0754717 |
| *Bosea* | sin+/+spu | 0 |
| *Bosea* | sin-/-spu | 0.96226415 |
| *Bosea* | sin+/-spu | 0.01886792 |
| *Bosea* | sin-/+spu | 0.01886792 |
| *Scardovia* | sin+/+spu | 0 |
| *Scardovia* | sin-/-spu | 0.69811321 |
| *Scardovia* | sin+/-spu | 0 |
| *Scardovia* | sin-/+spu | 0.30188679 |
| *Dialister* | sin+/+spu | 0 |
| *Dialister* | sin-/-spu | 0.86792453 |
| *Dialister* | sin+/-spu | 0 |
| *Dialister* | sin-/+spu | 0.13207547 |
| *Anaerococcus* | sin+/+spu | 0 |
| *Anaerococcus* | sin-/-spu | 0.9245283 |
| *Anaerococcus* | sin+/-spu | 0.05660377 |
| *Anaerococcus* | sin-/+spu | 0.01886792 |
| *Mogibacterium* | sin+/+spu | 0 |
| *Mogibacterium* | sin-/-spu | 0.81132075 |
| *Mogibacterium* | sin+/-spu | 0 |
| *Mogibacterium* | sin-/+spu | 0.18867925 |
| *Oribacterium* | sin+/+spu | 0 |
| *Oribacterium* | sin-/-spu | 0.79245283 |
| *Oribacterium* | sin+/-spu | 0 |
| *Oribacterium* | sin-/+spu | 0.20754717 |
| *Asinibacterium* | sin+/+spu | 0 |
| *Asinibacterium* | sin-/-spu | 0.83018868 |
| *Asinibacterium* | sin+/-spu | 0.09433962 |
| *Asinibacterium* | sin-/+spu | 0.0754717 |
| *Rhodococcus* | sin+/+spu | 0 |
| *Rhodococcus* | sin-/-spu | 0.98113208 |
| *Rhodococcus* | sin+/-spu | 0.01886792 |
| *Rhodococcus* | sin-/+spu | 0 |
| *Kingella* | sin+/+spu | 0 |
| *Kingella* | sin-/-spu | 0.9245283 |
| *Kingella* | sin+/-spu | 0 |
| *Kingella* | sin-/+spu | 0.0754717 |
| *Veillonellaceae* | sin+/+spu | 0 |
| *Veillonellaceae* | sin-/-spu | 0.98113208 |
| *Veillonellaceae* | sin+/-spu | 0 |
| *Veillonellaceae* | sin-/+spu | 0.01886792 |
| *Atopobium* | sin+/+spu | 0 |
| *Atopobium* | sin-/-spu | 0.66037736 |
| *Atopobium* | sin+/-spu | 0 |
| *Atopobium* | sin-/+spu | 0.33962264 |
| *Lactobacillus* | sin+/+spu | 0 |
| *Lactobacillus* | sin-/-spu | 0.81132075 |
| *Lactobacillus* | sin+/-spu | 0 |
| *Lactobacillus* | sin-/+spu | 0.18867925 |
| *Stomatobaculum* | sin+/+spu | 0 |
| *Stomatobaculum* | sin-/-spu | 0.90566038 |
| *Stomatobaculum* | sin+/-spu | 0 |
| *Stomatobaculum* | sin-/+spu | 0.09433962 |
| TM7x | sin+/+spu | 0 |
| TM7x | sin-/-spu | 0.86792453 |
| TM7x | sin+/-spu | 0 |
| TM7x | sin-/+spu | 0.13207547 |
| *Lautropia* | sin+/+spu | 0 |
| *Lautropia* | sin-/-spu | 0.96226415 |
| *Lautropia* | sin+/-spu | 0 |
| *Lautropia* | sin-/+spu | 0.03773585 |
| *Eubacteriumnodatugroup* | sin+/+spu | 0 |
| *Eubacteriumnodatugroup* | sin-/-spu | 0.88679245 |
| *Eubacteriumnodatugroup* | sin+/-spu | 0 |
| *Eubacteriumnodatugroup* | sin-/+spu | 0.11320755 |
| *Bulleidia* | sin+/+spu | 0 |
| *Bulleidia* | sin-/-spu | 0.98113208 |
| *Bulleidia* | sin+/-spu | 0 |
| *Bulleidia* | sin-/+spu | 0.01886792 |
| *Mitochondria* | sin+/+spu | 0 |
| *Mitochondria* | sin-/-spu | 0.98113208 |
| *Mitochondria* | sin+/-spu | 0 |
| *Mitochondria* | sin-/+spu | 0.01886792 |
| *Campylobacter* | sin+/+spu | 0 |
| *Campylobacter* | sin-/-spu | 0.79245283 |
| *Campylobacter* | sin+/-spu | 0 |
| *Campylobacter* | sin-/+spu | 0.20754717 |
| *Chryseobacterium* | sin+/+spu | 0 |
| *Chryseobacterium* | sin-/-spu | 0.98113208 |
| *Chryseobacterium* | sin+/-spu | 0 |
| *Chryseobacterium* | sin-/+spu | 0.01886792 |
| *Megasphaera* | sin+/+spu | 0 |
| *Megasphaera* | sin-/-spu | 0.86792453 |
| *Megasphaera* | sin+/-spu | 0 |
| *Megasphaera* | sin-/+spu | 0.13207547 |
| *Sphingomonas* | sin+/+spu | 0 |
| *Sphingomonas* | sin-/-spu | 0.94339623 |
| *Sphingomonas* | sin+/-spu | 0.03773585 |
| *Sphingomonas* | sin-/+spu | 0.01886792 |
| *Capnocytophaga* | sin+/+spu | 0.01886792 |
| *Capnocytophaga* | sin-/-spu | 0.77358491 |
| *Capnocytophaga* | sin+/-spu | 0 |
| *Capnocytophaga* | sin-/+spu | 0.20754717 |
| *Abiotrophia* | sin+/+spu | 0 |
| *Abiotrophia* | sin-/-spu | 0.79245283 |
| *Abiotrophia* | sin+/-spu | 0 |
| *Abiotrophia* | sin-/+spu | 0.20754717 |
| *Actinobacillus* | sin+/+spu | 0 |
| *Actinobacillus* | sin-/-spu | 0.94339623 |
| *Actinobacillus* | sin+/-spu | 0 |
| *Actinobacillus* | sin-/+spu | 0.05660377 |
| *Selenomonas* | sin+/+spu | 0 |
| *Selenomonas* | sin-/-spu | 0.81132075 |
| *Selenomonas* | sin+/-spu | 0 |
| *Selenomonas* | sin-/+spu | 0.18867925 |
| *Eikenella* | sin+/+spu | 0 |
| *Eikenella* | sin-/-spu | 0.94339623 |
| *Eikenella* | sin+/-spu | 0.01886792 |
| *Eikenella* | sin-/+spu | 0.03773585 |
| *Flavobacterium* | sin+/+spu | 0 |
| *Flavobacterium* | sin-/-spu | 0.98113208 |
| *Flavobacterium* | sin+/-spu | 0 |
| *Flavobacterium* | sin-/+spu | 0.01886792 |
| *Bradyrhizobium* | sin+/+spu | 0 |
| *Bradyrhizobium* | sin-/-spu | 0.98113208 |
| *Bradyrhizobium* | sin+/-spu | 0.01886792 |
| *Bradyrhizobium* | sin-/+spu | 0 |
| *Peptostreptococcus* | sin+/+spu | 0 |
| *Peptostreptococcus* | sin-/-spu | 0.88679245 |
| *Peptostreptococcus* | sin+/-spu | 0 |
| *Peptostreptococcus* | sin-/+spu | 0.11320755 |
| uncultured | sin+/+spu | 0 |
| uncultured | sin-/-spu | 0.98113208 |
| uncultured | sin+/-spu | 0 |
| uncultured | sin-/+spu | 0.01886792 |
| *Cardiobacterium* | sin+/+spu | 0 |
| *Cardiobacterium* | sin-/-spu | 0.98113208 |
| *Cardiobacterium* | sin+/-spu | 0 |
| *Cardiobacterium* | sin-/+spu | 0.01886792 |
| Unassigned | sin+/+spu | 0 |
| Unassigned | sin-/-spu | 0.86792453 |
| Unassigned | sin+/-spu | 0 |
| Unassigned | sin-/+spu | 0.13207547 |
| *Tepidimonas* | sin+/+spu | 0 |
| *Tepidimonas* | sin-/-spu | 0.9245283 |
| *Tepidimonas* | sin+/-spu | 0.01886792 |
| *Tepidimonas* | sin-/+spu | 0.05660377 |
| *Saccharimonadaceae* | sin+/+spu | 0 |
| *Saccharimonadaceae* | sin-/-spu | 0.96226415 |
| *Saccharimonadaceae* | sin+/-spu | 0 |
| *Saccharimonadaceae* | sin-/+spu | 0.03773585 |
| *Treponema* | sin+/+spu | 0 |
| *Treponema* | sin-/-spu | 0.98113208 |
| *Treponema* | sin+/-spu | 0 |
| *Treponema* | sin-/+spu | 0.01886792 |
| *Catonella* | sin+/+spu | 0 |
| *Catonella* | sin-/-spu | 0.90566038 |
| *Catonella* | sin+/-spu | 0 |
| *Catonella* | sin-/+spu | 0.09433962 |
| *Shuttleworthia* | sin+/+spu | 0 |
| *Shuttleworthia* | sin-/-spu | 0.96226415 |
| *Shuttleworthia* | sin+/-spu | 0 |
| *Shuttleworthia* | sin-/+spu | 0.03773585 |
| *Granulicatella* | sin+/+spu | 0 |
| *Granulicatella* | sin-/-spu | 0.98113208 |
| *Granulicatella* | sin+/-spu | 0 |
| *Granulicatella* | sin-/+spu | 0.01886792 |
| *Acinetobacter* | sin+/+spu | 0 |
| *Acinetobacter* | sin-/-spu | 0.98113208 |
| *Acinetobacter* | sin+/-spu | 0 |
| *Acinetobacter* | sin-/+spu | 0.01886792 |
| *Centipeda* | sin+/+spu | 0 |
| *Centipeda* | sin-/-spu | 0.98113208 |
| *Centipeda* | sin+/-spu | 0 |
| *Centipeda* | sin-/+spu | 0.01886792 |
| *Dolosigranulum* | sin+/+spu | 0.01886792 |
| *Dolosigranulum* | sin-/-spu | 0.83018868 |
| *Dolosigranulum* | sin+/-spu | 0.01886792 |
| *Dolosigranulum* | sin-/+spu | 0.13207547 |
| *Bradyrhizobium* | sin+/+spu | 0 |
| *Bradyrhizobium* | sin-/-spu | 0.9245283 |
| *Bradyrhizobium* | sin+/-spu | 0.01886792 |
| *Bradyrhizobium* | sin-/+spu | 0.05660377 |
| *Alloscardovia* | sin+/+spu | 0 |
| *Alloscardovia* | sin-/-spu | 0.96226415 |
| *Alloscardovia* | sin+/-spu | 0 |
| *Alloscardovia* | sin-/+spu | 0.03773585 |
| *Aggregatibacter* | sin+/+spu | 0 |
| *Aggregatibacter* | sin-/-spu | 0.98113208 |
| *Aggregatibacter* | sin+/-spu | 0 |
| *Aggregatibacter* | sin-/+spu | 0.01886792 |
| *Bergeyella* | sin+/+spu | 0 |
| *Bergeyella* | sin-/-spu | 0.88679245 |
| *Bergeyella* | sin+/-spu | 0 |
| *Bergeyella* | sin-/+spu | 0.11320755 |
| *Bacillus* | sin+/+spu | 0.05660377 |
| *Bacillus* | sin-/-spu | 0.52830189 |
| *Bacillus* | sin+/-spu | 0.11320755 |
| *Bacillus* | sin-/+spu | 0.30188679 |
| *Slackia* | sin+/+spu | 0 |
| *Slackia* | sin-/-spu | 0.98113208 |
| *Slackia* | sin+/-spu | 0 |
| *Slackia* | sin-/+spu | 0.01886792 |
| *Butyrivibrio* | sin+/+spu | 0 |
| *Butyrivibrio* | sin-/-spu | 0.98113208 |
| *Butyrivibrio* | sin+/-spu | 0 |
| *Butyrivibrio* | sin-/+spu | 0.01886792 |
| *Parvimonas* | sin+/+spu | 0 |
| *Parvimonas* | sin-/-spu | 0.94339623 |
| *Parvimonas* | sin+/-spu | 0 |
| *Parvimonas* | sin-/+spu | 0.05660377 |
| *Johnsonella* | sin+/+spu | 0 |
| *Johnsonella* | sin-/-spu | 0.98113208 |
| *Johnsonella* | sin+/-spu | 0 |
| *Johnsonella* | sin-/+spu | 0.01886792 |
| *Phreatobacter* | sin+/+spu | 0 |
| *Phreatobacter* | sin-/-spu | 0.96226415 |
| *Phreatobacter* | sin+/-spu | 0.01886792 |
| *Phreatobacter* | sin-/+spu | 0.01886792 |
| *Pseudoalteromonas* | sin+/+spu | 0.16981132 |
| *Pseudoalteromonas* | sin-/-spu | 0.45283019 |
| *Pseudoalteromonas* | sin+/-spu | 0.20754717 |
| *Pseudoalteromonas* | sin-/+spu | 0.16981132 |
| *Peptoniphilus* | sin+/+spu | 0 |
| *Peptoniphilus* | sin-/-spu | 0.96226415 |
| *Peptoniphilus* | sin+/-spu | 0.01886792 |
| *Peptoniphilus* | sin-/+spu | 0.01886792 |
| *Cryptobacterium* | sin+/+spu | 0 |
| *Cryptobacterium* | sin-/-spu | 0.98113208 |
| *Cryptobacterium* | sin+/-spu | 0 |
| *Cryptobacterium* | sin-/+spu | 0.01886792 |
| *Roseomonas* | sin+/+spu | 0 |
| *Roseomonas* | sin-/-spu | 0.98113208 |
| *Roseomonas* | sin+/-spu | 0 |
| *Roseomonas* | sin-/+spu | 0.01886792 |
| *Idiomarina* | sin+/+spu | 0 |
| *Idiomarina* | sin-/-spu | 0.96226415 |
| *Idiomarina* | sin+/-spu | 0.01886792 |
| *Idiomarina* | sin-/+spu | 0.01886792 |
| *Halomonas* | sin+/+spu | 0.01886792 |
| *Halomonas* | sin-/-spu | 0.86792453 |
| *Halomonas* | sin+/-spu | 0.05660377 |
| *Halomonas* | sin-/+spu | 0.05660377 |
| *Bifidobacterium* | sin+/+spu | 0 |
| *Bifidobacterium* | sin-/-spu | 0.98113208 |
| *Bifidobacterium* | sin+/-spu | 0 |
| *Bifidobacterium* | sin-/+spu | 0.01886792 |
| *Moraxella* | sin+/+spu | 0 |
| *Moraxella* | sin-/-spu | 0.98113208 |
| *Moraxella* | sin+/-spu | 0 |
| *Moraxella* | sin-/+spu | 0.01886792 |
| *Tessaracoccus* | sin+/+spu | 0 |
| *Tessaracoccus* | sin-/-spu | 1 |
| *Tessaracoccus* | sin+/-spu | 0 |
| *Tessaracoccus* | sin-/+spu | 0 |
| *Arthrobacter* | sin+/+spu | 0 |
| *Arthrobacter* | sin-/-spu | 1 |
| *Arthrobacter* | sin+/-spu | 0 |
| *Arthrobacter* | sin-/+spu | 0 |
| *Rhodopseudomonas* | sin+/+spu | 0 |
| *Rhodopseudomonas* | sin-/-spu | 1 |
| *Rhodopseudomonas* | sin+/-spu | 0 |
| *Rhodopseudomonas* | sin-/+spu | 0 |
| *Pandoraea* | sin+/+spu | 0 |
| *Pandoraea* | sin-/-spu | 1 |
| *Pandoraea* | sin+/-spu | 0 |
| *Pandoraea* | sin-/+spu | 0 |
| *Cloacibacterium* | sin+/+spu | 0 |
| *Cloacibacterium* | sin-/-spu | 1 |
| *Cloacibacterium* | sin+/-spu | 0 |
| *Cloacibacterium* | sin-/+spu | 0 |
| *Paenibacillus* | sin+/+spu | 0 |
| *Paenibacillus* | sin-/-spu | 1 |
| *Paenibacillus* | sin+/-spu | 0 |
| *Paenibacillus* | sin-/+spu | 0 |
| *Exiguobacterium* | sin+/+spu | 0 |
| *Exiguobacterium* | sin-/-spu | 1 |
| *Exiguobacterium* | sin+/-spu | 0 |
| *Exiguobacterium* | sin-/+spu | 0 |
| *Klebsiella* | sin+/+spu | 0 |
| *Klebsiella* | sin-/-spu | 1 |
| *Klebsiella* | sin+/-spu | 0 |
| *Klebsiella* | sin-/+spu | 0 |
| *Janthinobacterium* | sin+/+spu | 0 |
| *Janthinobacterium* | sin-/-spu | 1 |
| *Janthinobacterium* | sin+/-spu | 0 |
| *Janthinobacterium* | sin-/+spu | 0 |
| *Eubacteriumbrachgroup* | sin+/+spu | 0 |
| *Eubacteriumbrachgroup* | sin-/-spu | 1 |
| *Eubacteriumbrachgroup* | sin+/-spu | 0 |
| *Eubacteriumbrachgroup* | sin-/+spu | 0 |
| *Sediminibacterium* | sin+/+spu | 0 |
| *Sediminibacterium* | sin-/-spu | 1 |
| *Sediminibacterium* | sin+/-spu | 0 |
| *Sediminibacterium* | sin-/+spu | 0 |
| *Oceanobacillus* | sin+/+spu | 0 |
| *Oceanobacillus* | sin-/-spu | 1 |
| *Oceanobacillus* | sin+/-spu | 0 |
| *Oceanobacillus* | sin-/+spu | 0 |
| *Pseudochrobactrum* | sin+/+spu | 0 |
| *Pseudochrobactrum* | sin-/-spu | 1 |
| *Pseudochrobactrum* | sin+/-spu | 0 |
| *Pseudochrobactrum* | sin-/+spu | 0 |
| *Tannerella* | sin+/+spu | 0 |
| *Tannerella* | sin-/-spu | 1 |
| *Tannerella* | sin+/-spu | 0 |
| *Tannerella* | sin-/+spu | 0 |
| *Elizabethkingia* | sin+/+spu | 0 |
| *Elizabethkingia* | sin-/-spu | 1 |
| *Elizabethkingia* | sin+/-spu | 0 |
| *Elizabethkingia* | sin-/+spu | 0 |
| *Acidovorax* | sin+/+spu | 0 |
| *Acidovorax* | sin-/-spu | 1 |
| *Acidovorax* | sin+/-spu | 0 |
| *Acidovorax* | sin-/+spu | 0 |
| *Weissella* | sin+/+spu | 0 |
| *Weissella* | sin-/-spu | 1 |
| *Weissella* | sin+/-spu | 0 |
| *Weissella* | sin-/+spu | 0 |
| *Fretibacterium* | sin+/+spu | 0 |
| *Fretibacterium* | sin-/-spu | 1 |
| *Fretibacterium* | sin+/-spu | 0 |
| *Fretibacterium* | sin-/+spu | 0 |
| *Delftia* | sin+/+spu | 0 |
| *Delftia* | sin-/-spu | 1 |
| *Delftia* | sin+/-spu | 0 |
| *Delftia* | sin-/+spu | 0 |
| 1174 | sin+/+spu | 0 |
| 1174 | sin-/-spu | 1 |
| 1174 | sin+/-spu | 0 |
| 1174 | sin-/+spu | 0 |
| *Parasediminibacterium* | sin+/+spu | 0 |
| *Parasediminibacterium* | sin-/-spu | 1 |
| *Parasediminibacterium* | sin+/-spu | 0 |
| *Parasediminibacterium* | sin-/+spu | 0 |
| *Hymenobacter* | sin+/+spu | 0 |
| *Hymenobacter* | sin-/-spu | 1 |
| *Hymenobacter* | sin+/-spu | 0 |
| *Hymenobacter* | sin-/+spu | 0 |
| *Enhydrobacter* | sin+/+spu | 0 |
| *Enhydrobacter* | sin-/-spu | 1 |
| *Enhydrobacter* | sin+/-spu | 0 |
| *Enhydrobacter* | sin-/+spu | 0 |
| *Mobiluncus* | sin+/+spu | 0 |
| *Mobiluncus* | sin-/-spu | 1 |
| *Mobiluncus* | sin+/-spu | 0 |
| *Mobiluncus* | sin-/+spu | 0 |
| *Salinarimonas* | sin+/+spu | 0 |
| *Salinarimonas* | sin-/-spu | 1 |
| *Salinarimonas* | sin+/-spu | 0 |
| *Salinarimonas* | sin-/+spu | 0 |
| uncultured | sin+/+spu | 0 |
| uncultured | sin-/-spu | 1 |
| uncultured | sin+/-spu | 0 |
| uncultured | sin-/+spu | 0 |
| *Eubacteriumyurigroup* | sin+/+spu | 0 |
| *Eubacteriumyurigroup* | sin-/-spu | 1 |
| *Eubacteriumyurigroup* | sin+/-spu | 0 |
| *Eubacteriumyurigroup* | sin-/+spu | 0 |
| *Hyphomicrobium* | sin+/+spu | 0 |
| *Hyphomicrobium* | sin-/-spu | 1 |
| *Hyphomicrobium* | sin+/-spu | 0 |
| *Hyphomicrobium* | sin-/+spu | 0 |
| *Roseisolibacter* | sin+/+spu | 0 |
| *Roseisolibacter* | sin-/-spu | 1 |
| *Roseisolibacter* | sin+/-spu | 0 |
| *Roseisolibacter* | sin-/+spu | 0 |
| *Cutibacterium* | sin+/+spu | 0 |
| *Cutibacterium* | sin-/-spu | 1 |
| *Cutibacterium* | sin+/-spu | 0 |
| *Cutibacterium* | sin-/+spu | 0 |
| *Comamonas* | sin+/+spu | 0 |
| *Comamonas* | sin-/-spu | 1 |
| *Comamonas* | sin+/-spu | 0 |
| *Comamonas* | sin-/+spu | 0 |
| *Brochothrix* | sin+/+spu | 0 |
| *Brochothrix* | sin-/-spu | 1 |
| *Brochothrix* | sin+/-spu | 0 |
| *Brochothrix* | sin-/+spu | 0 |
| F0058 | sin+/+spu | 0 |
| F0058 | sin-/-spu | 1 |
| F0058 | sin+/-spu | 0 |
| F0058 | sin-/+spu | 0 |
| *Serratia* | sin+/+spu | 0 |
| *Serratia* | sin-/-spu | 1 |
| *Serratia* | sin+/-spu | 0 |
| *Serratia* | sin-/+spu | 0 |
| *Micrococcus* | sin+/+spu | 0 |
| *Micrococcus* | sin-/-spu | 1 |
| *Micrococcus* | sin+/-spu | 0 |
| *Micrococcus* | sin-/+spu | 0 |
| *Brevundimonas* | sin+/+spu | 0 |
| *Brevundimonas* | sin-/-spu | 1 |
| *Brevundimonas* | sin+/-spu | 0 |
| *Brevundimonas* | sin-/+spu | 0 |
| *Afipia* | sin+/+spu | 0 |
| *Afipia* | sin-/-spu | 1 |
| *Afipia* | sin+/-spu | 0 |
| *Afipia* | sin-/+spu | 0 |
| *CandidatuSaccharimonas* | sin+/+spu | 0 |
| *CandidatuSaccharimonas* | sin-/-spu | 1 |
| *CandidatuSaccharimonas* | sin+/-spu | 0 |
| *CandidatuSaccharimonas* | sin-/+spu | 0 |
