# Supplementary material for: Cystic fibrosis pathogens persist in the upper respiratory tract following initiation of elexacaftor/tezacaftor/ivacaftor therapy
Source: Microbiol Spectr. 2024 Jun 25;12(8):e00787-24. doi: 10.1128/spectrum.00787-24 (PMC11302335; doi:10.1128/spectrum.00787-24)
Supplement: Table S3 — Concordance metrics per genera for paired sinus and sputum samples. [file spectrum.00787-24-s0007.docx]

Table S3: Full table of concordance metrics per genera for sinus and sputum microbiome comparisons. Sensitivity = sin+/+spu / (sin+/+spu + sin-/+spu). Specificity = sin-/-spu / (sin-/-spu + sin+/-spu). Precision = sin+/+spu / (sin+/+spu + sin+/-spu). Accuracy = (sin+/+spu + sin-/-spu) / total. F measure = (2 x sensitivity x precision) / (sensitivity + precision).

| ***genus*** | **sensitivity** | **specificity** | **precision** | **accuracy** | **F_measure** |
| --- | --- | --- | --- | --- | --- |
| 1174 | NA | 1 | NA | 1 | NA |
| *Abiotrophia* | 0 | 1 | NA | 0.79245283 | NA |
| *Achromobacter* | 0.46153846 | 0.75 | 0.375 | 0.67924528 | 0.4137931 |
| *Acidovorax* | NA | 1 | NA | 1 | NA |
| *Acinetobacter* | 0 | 1 | NA | 0.98113208 | NA |
| *Actinobacillus* | 0 | 1 | NA | 0.94339623 | NA |
| *Actinomyces* | 0.02272727 | 1 | 1 | 0.18867925 | 0.04444444 |
| *Afipia* | NA | 1 | NA | 1 | NA |
| *Aggregatibacter* | 0 | 1 | NA | 0.98113208 | NA |
| *Alloprevotella* | 0 | 1 | NA | 0.81132075 | NA |
| *Alloscardovia* | 0 | 1 | NA | 0.96226415 | NA |
| *Anaerococcus* | 0 | 0.94230769 | 0 | 0.9245283 | NA |
| *Arthrobacter* | NA | 1 | NA | 1 | NA |
| *Asinibacterium* | 0 | 0.89795918 | 0 | 0.83018868 | NA |
| *Atopobium* | 0 | 1 | NA | 0.66037736 | NA |
| *Bacillus* | 0.15789474 | 0.82352941 | 0.33333333 | 0.58490566 | 0.21428571 |
| *Bergeyella* | 0 | 1 | NA | 0.88679245 | NA |
| *Bifidobacterium* | 0 | 1 | NA | 0.98113208 | NA |
| *Bosea* | 0 | 0.98076923 | 0 | 0.96226415 | NA |
| *Bradyrhizobium* | 0 | 0.98058252 | 0 | 0.95283019 | NA |
| *Brevundimonas* | NA | 1 | NA | 1 | NA |
| *Brochothrix* | NA | 1 | NA | 1 | NA |
| *Bulleidia* | 0 | 1 | NA | 0.98113208 | NA |
| *Burkholderia* | 0 | 0.96153846 | 0 | 0.94339623 | NA |
| *Butyrivibrio* | 0 | 1 | NA | 0.98113208 | NA |
| *Campylobacter* | 0 | 1 | NA | 0.79245283 | NA |
| *CandidatuSaccharimonas* | NA | 1 | NA | 1 | NA |
| *Capnocytophaga* | 0.08333333 | 1 | 1 | 0.79245283 | 0.15384615 |
| *Cardiobacterium* | 0 | 1 | NA | 0.98113208 | NA |
| *Catonella* | 0 | 1 | NA | 0.90566038 | NA |
| *Centipeda* | 0 | 1 | NA | 0.98113208 | NA |
| *Chryseobacterium* | 0 | 1 | NA | 0.98113208 | NA |
| *Cloacibacterium* | NA | 1 | NA | 1 | NA |
| *Comamonas* | NA | 1 | NA | 1 | NA |
| *Corynebacterium* | 0.36363636 | 0.67741935 | 0.44444444 | 0.54716981 | 0.4 |
| *Cryptobacterium* | 0 | 1 | NA | 0.98113208 | NA |
| *Cutibacterium* | NA | 1 | NA | 1 | NA |
| *Delftia* | NA | 1 | NA | 1 | NA |
| *Dialister* | 0 | 1 | NA | 0.86792453 | NA |
| *Dolosigranulum* | 0.125 | 0.97777778 | 0.5 | 0.8490566 | 0.2 |
| *Eikenella* | 0 | 0.98039216 | 0 | 0.94339623 | NA |
| *Elizabethkingia* | NA | 1 | NA | 1 | NA |
| *Enhydrobacter* | NA | 1 | NA | 1 | NA |
| *Enterococcus* | 0 | 1 | NA | 0.94339623 | NA |
| *Escherichia* | 0.4 | 0.69767442 | 0.23529412 | 0.64150943 | 0.2962963 |
| *Eubacteriumbrachgroup* | NA | 1 | NA | 1 | NA |
| *Eubacteriumnodatugroup* | 0 | 1 | NA | 0.88679245 | NA |
| *Eubacteriumyurigroup* | NA | 1 | NA | 1 | NA |
| *Exiguobacterium* | NA | 1 | NA | 1 | NA |
| F0058 | NA | 1 | NA | 1 | NA |
| *Finegoldia* | 0 | 0.89795918 | 0 | 0.83018868 | NA |
| *Flavobacterium* | 0 | 1 | NA | 0.98113208 | NA |
| *Fretibacterium* | NA | 1 | NA | 1 | NA |
| *Fusobacterium* | 0 | 1 | NA | 0.64150943 | NA |
| *Gemella* | 0.05555556 | 1 | 1 | 0.35849057 | 0.10526316 |
| *Granulicatella* | 0.07407407 | 0.97468354 | 0.5 | 0.74528302 | 0.12903226 |
| *Haemophilus* | 0.10344828 | 0.93506494 | 0.375 | 0.70754717 | 0.16216216 |
| *Halomonas* | 0.25 | 0.93877551 | 0.25 | 0.88679245 | 0.25 |
| *Hymenobacter* | NA | 1 | NA | 1 | NA |
| *Hyphomicrobium* | NA | 1 | NA | 1 | NA |
| *Idiomarina* | 0 | 0.98076923 | 0 | 0.96226415 | NA |
| *Janthinobacterium* | NA | 1 | NA | 1 | NA |
| *Johnsonella* | 0 | 1 | NA | 0.98113208 | NA |
| *Kingella* | 0 | 1 | NA | 0.9245283 | NA |
| *Klebsiella* | NA | 1 | NA | 1 | NA |
| *Lachnoanaerobaculum* | 0 | 1 | NA | 0.71698113 | NA |
| *Lactobacillus* | 0 | 1 | NA | 0.81132075 | NA |
| *Lautropia* | 0 | 1 | NA | 0.96226415 | NA |
| *Lawsonella* | 0.25 | 0.91836735 | 0.2 | 0.86792453 | 0.22222222 |
| *Leptotrichia* | 0 | 1 | NA | 0.81132075 | NA |
| *Megasphaera* | 0 | 1 | NA | 0.86792453 | NA |
| *Mesorhizobium* | NA | 0.98113208 | 0 | 0.98113208 | NA |
| *Methylobacterium* | 0 | 0.96153846 | 0 | 0.94339623 | NA |
| *Micrococcus* | NA | 1 | NA | 1 | NA |
| *Mitochondria* | 0 | 1 | NA | 0.98113208 | NA |
| *Mobiluncus* | NA | 1 | NA | 1 | NA |
| *Mogibacterium* | 0 | 1 | NA | 0.81132075 | NA |
| *Moraxella* | 0 | 1 | NA | 0.98113208 | NA |
| *Neisseria* | 0 | 0.91428571 | 0 | 0.60377358 | NA |
| *Oceanobacillus* | NA | 1 | NA | 1 | NA |
| *Oribacterium* | 0 | 1 | NA | 0.79245283 | NA |
| *Paenibacillus* | NA | 1 | NA | 1 | NA |
| *Pandoraea* | 0 | 0.99029126 | 0 | 0.96226415 | NA |
| *Parasediminibacterium* | NA | 1 | NA | 1 | NA |
| *Parvimonas* | 0 | 1 | NA | 0.94339623 | NA |
| *Peptoniphilus* | 0 | 0.98076923 | 0 | 0.96226415 | NA |
| *Peptostreptococcus* | 0 | 1 | NA | 0.88679245 | NA |
| *Phreatobacter* | 0 | 0.98076923 | 0 | 0.96226415 | NA |
| *Porphyromonas* | 0 | 1 | NA | 0.73584906 | NA |
| *Prevotella* | 0.03225806 | 1 | 1 | 0.43396226 | 0.0625 |
| *Pseudoalteromonas* | 0.5 | 0.68571429 | 0.45 | 0.62264151 | 0.47368421 |
| *Pseudochrobactrum* | NA | 1 | NA | 1 | NA |
| *Pseudomonas* | 0.95918367 | 0 | 0.92156863 | 0.88679245 | 0.94 |
| *Reyranella* | 0 | 0.92307692 | 0 | 0.90566038 | NA |
| *Rhodococcus* | NA | 0.98113208 | 0 | 0.98113208 | NA |
| *Rhodopseudomonas* | NA | 1 | NA | 1 | NA |
| *Roseisolibacter* | NA | 1 | NA | 1 | NA |
| *Roseomonas* | 0 | 1 | NA | 0.98113208 | NA |
| *Rothia* | 0.29787234 | 0.66666667 | 0.875 | 0.33962264 | 0.44444444 |
| *Saccharimonadaceae* | 0 | 1 | NA | 0.96226415 | NA |
| *Salinarimonas* | NA | 1 | NA | 1 | NA |
| *Scardovia* | 0 | 1 | NA | 0.69811321 | NA |
| *Sediminibacterium* | NA | 1 | NA | 1 | NA |
| *Selenomonas* | 0 | 1 | NA | 0.81132075 | NA |
| *Serratia* | NA | 1 | NA | 1 | NA |
| *Shuttleworthia* | 0 | 1 | NA | 0.96226415 | NA |
| *Slackia* | 0 | 1 | NA | 0.98113208 | NA |
| *Solobacterium* | 0 | 0.97058824 | 0 | 0.62264151 | NA |
| *Sphingomonas* | 0 | 0.96153846 | 0 | 0.94339623 | NA |
| *Staphylococcus* | 0.96226415 | NA | 1 | 0.96226415 | 0.98076923 |
| *Stenotrophomonas* | 0 | 0.98 | 0 | 0.9245283 | NA |
| *Stomatobaculum* | 0 | 1 | NA | 0.90566038 | NA |
| *Streptococcus* | 0.67307692 | 0 | 0.97222222 | 0.66037736 | 0.79545455 |
| TM7x | 0 | 1 | NA | 0.86792453 | NA |
| *Tannerella* | NA | 1 | NA | 1 | NA |
| *Tepidimonas* | 0 | 0.98 | 0 | 0.9245283 | NA |
| *Tessaracoccus* | NA | 1 | NA | 1 | NA |
| *Treponema* | 0 | 1 | NA | 0.98113208 | NA |
| Unassigned | 0 | 1 | NA | 0.86792453 | NA |
| *Veillonella* | 0.14285714 | 0.88888889 | 0.71428571 | 0.39622642 | 0.23809524 |
| *Veillonellaceae* | 0 | 1 | NA | 0.98113208 | NA |
| *Weissella* | NA | 1 | NA | 1 | NA |
| uncultured | 0 | 1 | NA | 0.99056604 | NA |
