# Supplementary material for: Cystic fibrosis pathogens persist in the upper respiratory tract following initiation of elexacaftor/tezacaftor/ivacaftor therapy
Source: Microbiol Spectr. 2024 Jun 25;12(8):e00787-24. doi: 10.1128/spectrum.00787-24 (PMC11302335; doi:10.1128/spectrum.00787-24)
Supplement: Table S4 — Custom oligos used. [file spectrum.00787-24-s0008.docx]

Table S4: Custom oligos amplified alongside V4 (515f – 806r) and ITS (ITS1f – ITS2) primers.

| **organism** | **Target** | **primer** | **name** | **sequence** | **length** | **Tm** | **GC** | **amplicon_length** |
| --- | --- | --- | --- | --- | --- | --- | --- | --- |
| Pseudomonas_aeruginosa | tseT | forward | Pa_tseT_F | GACCAATTTCCCGTTTC | 17 | 50 | 47.1 | 381 |
| Pseudomonas_aeruginosa | tseT | reverse | Pa_tseT_R | ATCTCTTCCTGTTCCTTC | 18 | 50 | 44.4 | 381 |
| Pseudomonas_aeruginosa | tsiT | forward | Pa_tsiT_F | CTGGAGAACTGGTATCTG | 18 | 50 | 50 | 351 |
| Pseudomonas_aeruginosa | tsiT | reverse | Pa_tsiT_R | CTGGTGTACGAAGTACG | 17 | 50 | 52.9 | 351 |
| Pseudomonas_aeruginosa_Gp1 | exoS | forward | Pa_exoS_F | GAGGTCAGCAGAGTATC | 17 | 55 | 52.9 | 273 |
| Pseudomonas_aeruginosa_Gp1 | exoS | reverse | Pa_exoS_R | AGTCTTCACTACCTGTTC | 18 | 55 | 44.4 | 273 |
| Pseudomonas_aeruginosa_Gp2 | exoU | forward | Pa_exoU_f | CCTGAGATGATCGACAA | 17 | 55 | 47.1 | 382 |
| Pseudomonas_aeruginosa_Gp2 | exoU | reverse | Pa_exoU_R | GAGAAGCGAAGGTATGA | 17 | 55 | 47.1 | 382 |
| Pseudomonas_aeruginosa_Gp2 | pelA | forward | Pa_pelA_F | TTCATCAAGCCCTATCC | 17 | 55 | 47.1 | 229 |
| Pseudomonas_aeruginosa_Gp2 | pelA | reverse | Pa_pelA_R | CCATCTTGTAGCCATACT | 18 | 56 | 44.4 | 229 |
| Pseudomonas_aeruginosa_Gp1 | pslA | forward | Pa_pslA_F | GATGTACACGGAAAGGA | 17 | 56 | 47.1 | 381 |
| Pseudomonas_aeruginosa_Gp1 | pslA | reverse | Pa_pslA_R | CGGAACAGGATGTAGAG | 17 | 55 | 52.9 | 381 |
| Staphylococcus_aureus_MRSA | mecA | forward | Sa_mecA_F | CATTAGAAGGTGAAGATCAA | 20 | 55 | 35 | 313 |
| Staphylococcus_aureus_MRSA | mecA | reverse | Sa_mecA_R | GTTCTGTCTGTTGGATAAG | 19 | 55 | 42.1 | 313 |
| Staphylococcus_epidermidis | gseA | forward | Se_gseA_F | TACCGTATCCTGGTAATAG | 19 | 55 | 42.1 | 212 |
| Staphylococcus_epidermidis | gseA | reverse | Se_gseA_R | CTCGCCACCAATATAGA | 17 | 55 | 47.1 | 212 |
| Staphylococcus_aureus | ldh1 | forward | Sa_ldh1_F | CTGGAGAAACACGTTTAG | 18 | 55 | 44.4 | 303 |
| Staphylococcus_aureus | ldh1 | reverse | Sa_ldh1_R | GTGACCATACTGGTAATTC | 19 | 55 | 42.1 | 303 |
| Pseudomonas_Pf5_phage | PA14_48930 | forward | Pf5_F | GATAGCAACTGGTGGTCTTC | 20 | 53 | 50 | 254 |
| Pseudomonas_Pf5_phage | PA14_48930 | reverse | Pf5_R | GCCGAATTTCACTATTTCCG | 20 | 52 | 45 | 254 |
